# Supplementary material for: Pharmacological activation of constitutive androstane receptor induces female-specific modulation of hepatic metabolism
Source: JHEP Rep. 2023 Oct 13;6(1):100930. doi: 10.1016/j.jhepr.2023.100930 (PMC10749885; doi:10.1016/j.jhepr.2023.100930)
Supplement: Multimedia component 1 [file mmc1.pdf]

Pharmacological activation of constitutive androstane receptor induces female-specific modulation of hepatic metabolism

Marine Huillet, Frédéric Lasserre, Marie-Pierre Gratacap, Beatrice Engelmann, Justine Bruse, Arnaud Polizzi, Tiffany Fougeray, Céline Martin, Clémence Rives, Anne Fougerat, Claire Naylies, Yannick Lippi, Géraldine Garcia, Elodie Rousseau-Bacquie, Cécile Canlet, Laurent Debrauwer, Ulrike Rolle-Kampczyk, Martin von Bergen, Bernard Payrastre, Elisa Boutet-Robinet, Laurence Gamet-Payrastre, Hervé Guillou, Nicolas Loiseau, Sandrine Ellero-Simatos

Table of contents

Supplementary methods…………………………………………………………….....2

Fig. S1……………………………………………………………………………………3

Fig. S2……………………………………………………………………………………4

Fig. S3……………………………………………………………………………………5

Fig. S4……………………………………………………………………………………6

Fig. S5……………………………………………………………………………………7

Fig. S6……………………………………………………………………………………8

Fig. S7……………………………………………………………………………………9

Table S1………………………………………………………………………………..10

Table S2………………………………………………………………………………..11

Table S3………………………………………………………………………………..13

Table S4-6………………………………………………………………separate excel files

Supplementary references……………………………………………………………..…17

Supplementary methods

1. Trimethylamine-N-oxide targeted LC-MS/MS measurement

For TMAO extraction method from [1] was modified. Liver tissue was combined with 425 µl ice-cold methanol and 75 µl 1% formic acid. Samples were homogenized using a TissueLyser II (30 Hz, 10 min; Retsch Qiagen). After centrifugation (5 min, 8°C, 14.000 rpm) 170 µl of supernatant was transferred to a new tube and evaporated to dryness (Eppendorf concentrator plus, Eppendorf). Prior to measurement, samples were resuspended in LC solvent A:B (1:1), volume depending on the liver weight used for extraction. Of each sample, 10 µl were injected onto an Agilent 1290 II infinity UPLC system (Agilent Technologies Inc., Santa Clara, USA) coupled online to a QTRAP®6500 mass spectrometer (Sciex, Framingham, USA). Chromatographic run was achieved using a reverse phase XSelect HSS T3 XP column (2.1 x 150 mm, 2.5 µm, 100 Å; Waters, Milford, USA) with a binary solvent system 5% acetonitrile with 0.1% formic acid in water (A) and 5% water with 0.1% formic acid in acetonitrile (B). Constant flow rate was set to 250 µl and column oven temperature was at 30°C. Mass spectrometric measurement was performed in positive ionization mode. For identification and quantitation, a scheduled multiple reaction monitoring (MRM) method was used with m/z 76.0 → 59.0 and m/z 75.0 → 42.2 as specific transitions for TMAO. Peak areas were determined in Analyst® Software (v. 1.7.1, Sciex) and exported. Analytical standard trimethylamine N-oxide (TMAO) was purchased from Sigma Aldrich (St Louis, MO, USA). All solvents for MS were of analytical grade purity. Experimental water (resistivity of 18.2 MΩ cm) was purified using a Milli-Q system (Millipore, Milford, MA, USA).

Fig. S1: Impact of CAR activation on hepatic transcripts in *Car*$^{+/+}$ *vs. Car*$^{-/-}$ mice. A) PCA score plots of the whole liver transcriptomic dataset in *Car*$^{+/+}$ and *Car*$^{-/-}$ mice (n=6/group). B) Venn diagram representing the number of genes significantly modulated by TCPOBOP in the liver of *Car*$^{-/-}$ *vs. Car*$^{+/+}$ male and female mice.

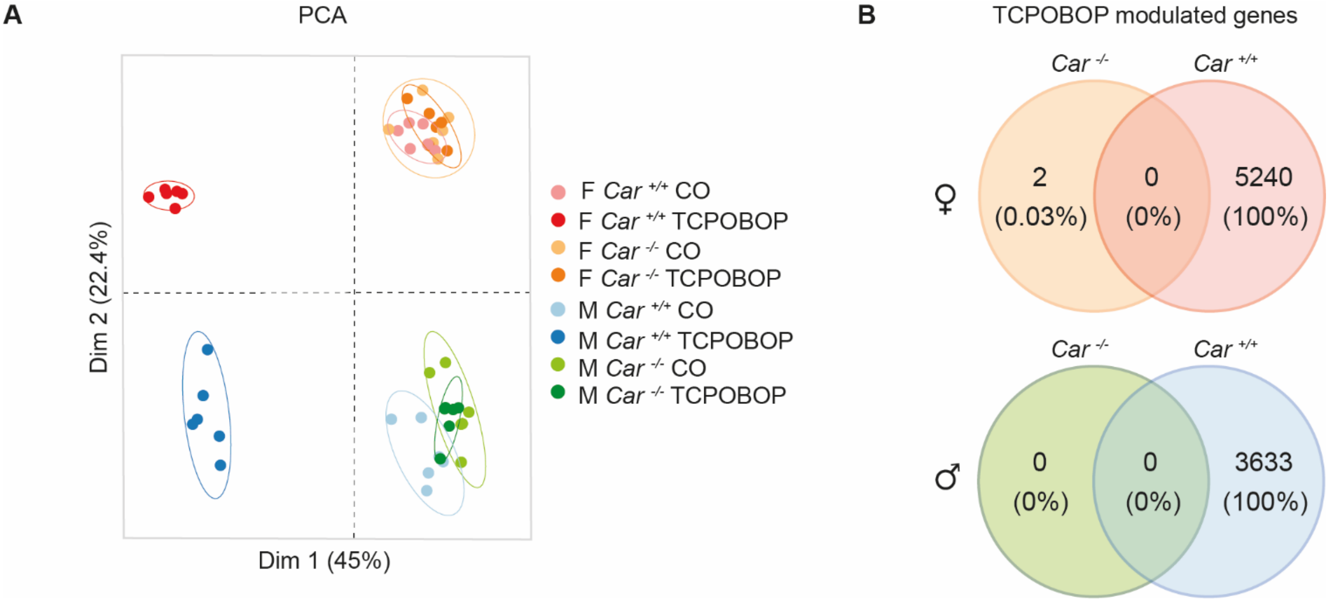

Fig. S2: Majority of TCPOBOP-modulated genes are common between sexes. A) Venn diagram representing the number of genes significantly modulated by TCPOBOP in *Car* $^{+/+}$ male and female mice liver considering significant regulation at P_{adj}<0.05 and 3 different fold-change thresholds (TCPOBOP *vs.* CO). B) Venn diagram representing the number of genes significantly modulated by TCPOBOP in *Car*$^{+/+}$ male and female mouse liver considering significant regulation at P_{adj}<0.01 and 3 different fold-change thresholds (TCPOBOP *vs.* CO).

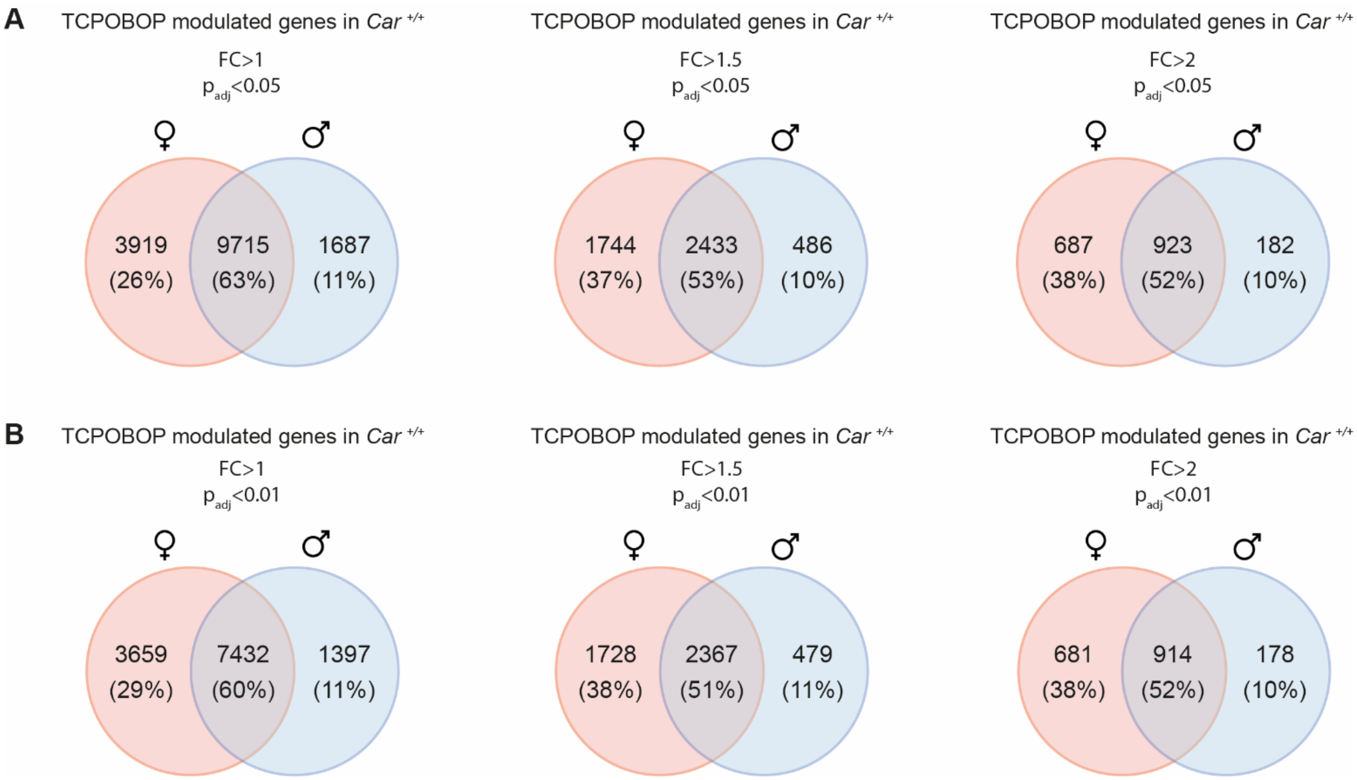

Fig. S3: ^{1}H-NMR-based metabolomics in plasma samples from *Car*$^{-/-}$ mice. A) Coefficient plots related to the PLS-DA models discriminating between ^{1}H-NMR-based plasma spectra from TCPOBOP *vs.* CO *Car*$^{-/-}$ males. Parameters of the PLS-DA model: $Q^2Y=0.1$, $p>0.05$. B) Coefficient plots related to the PLS-DA models discriminating between ^{1}H-NMR-based plasma spectra from TCPOBOP *vs.* CO *Car*$^{-/-}$ females. Parameters of the PLS-DA model: $Q^2Y=-0.4$, $p>0.05$.

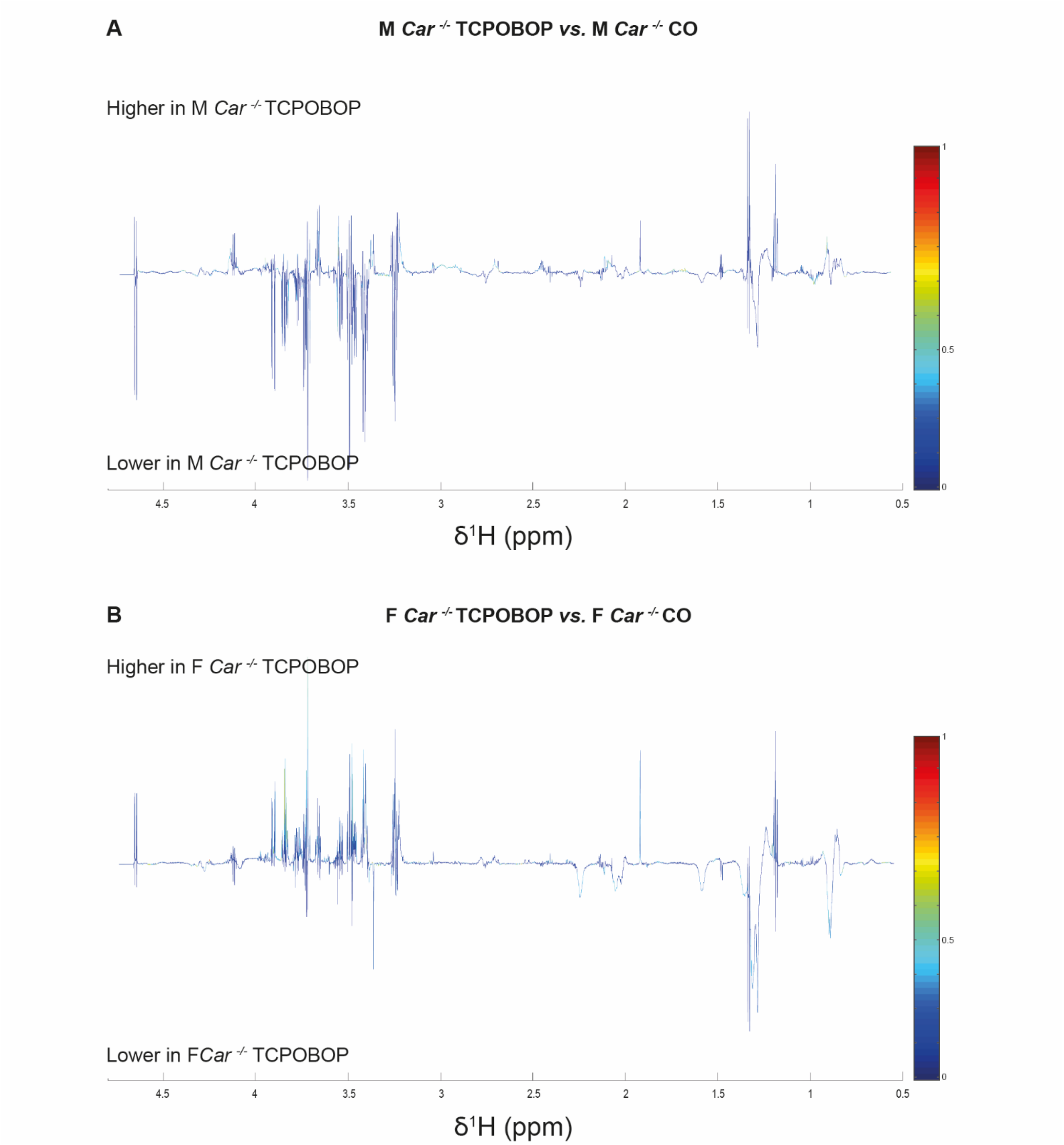

Fig. S4: Effect of CAR activation on plasma biochemistry. A) Plasma cholesterol concentration was determined on plasma collected at the end of the experiment. B) Plasma concentration of high-density lipoprotein (HDL) was determined on plasma collected at the end of the experiment. C) Plasma concentration of triglycerides was determined on plasma collected at the end of the experiment. D) Plasma concentration of free fatty acids was determined on plasma collected at the end of the experiment. E) Plasma concentration of glucose was determined on plasma collected at the end of the experiment. Results are given as the mean ± SEM. *treatment effect, #sex effect. * or # $p<0.05$, ** or ## $p<0.01$, *** or ### $p<0.001$.

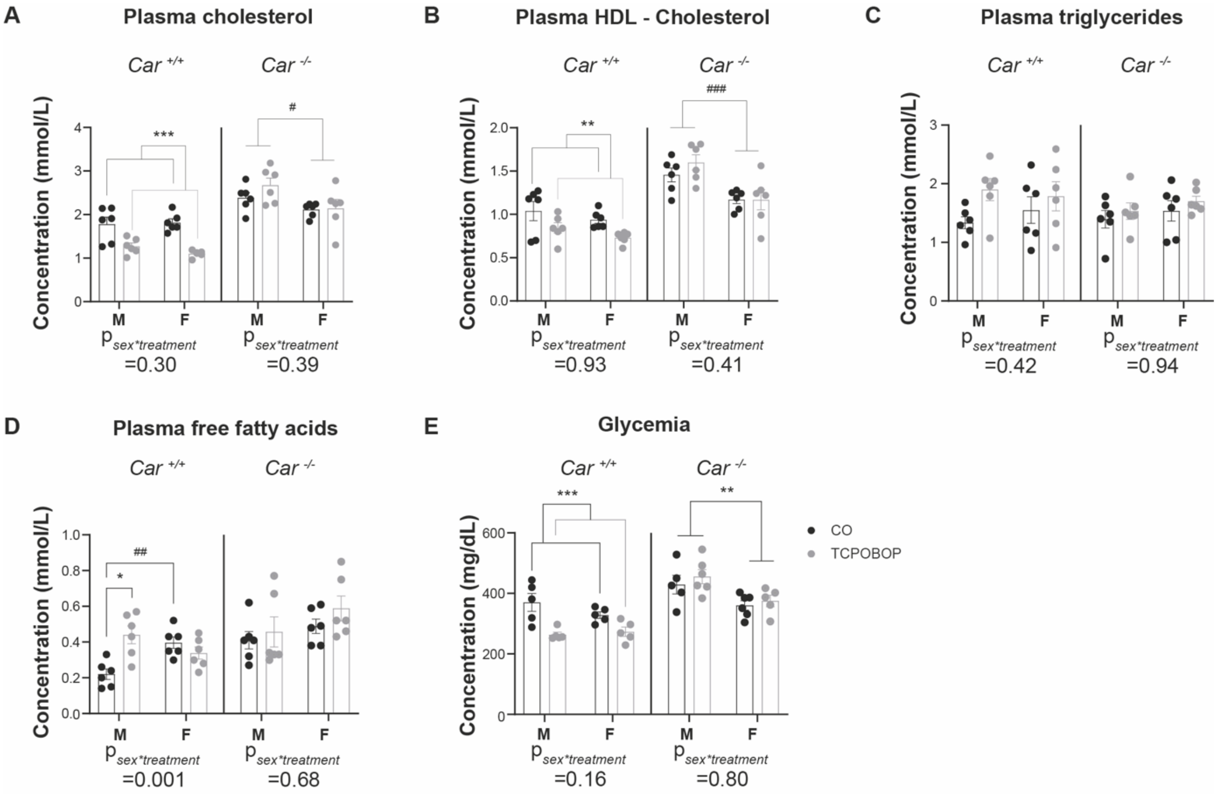

Fig. S5: Effect of CAR activation on hepatic mRNA expression of genes involved in lipoprotein metabolism in *Car$^{-/-}$* mice. A) Fold-change (TCPOBOP- *vs.* CO-treated *Car$^{-/-}$* mice) of hepatic expression for genes involved in lipoprotein metabolism (derived from microarray results).

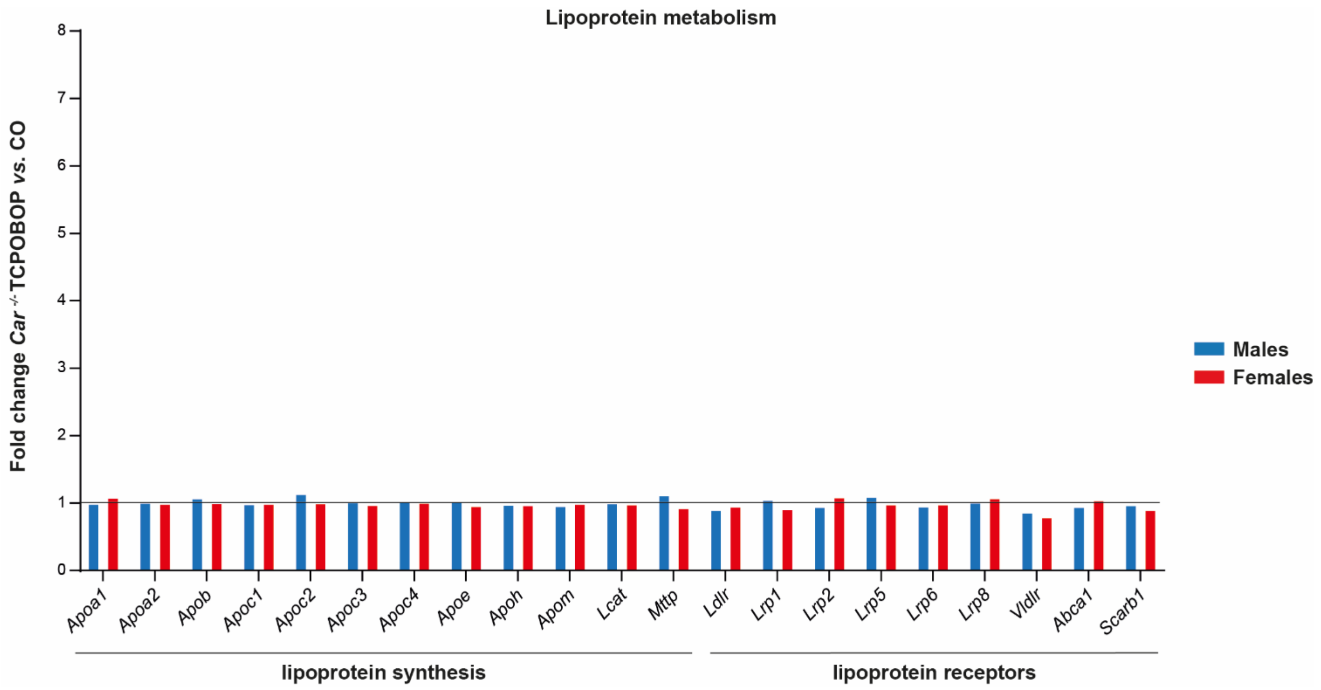

Fig. S6: Pharmacological activation of CAR modulates expression of genes involved in bile acid metabolism and cholesterol metabolism. A) Fold-change (TCPOBOP- *vs.* CO-treated $Car^{+/+}$ mice) of hepatic expression for genes involved in bile acid metabolism (derived from microarray results). B) Fold-change (TCPOBOP- *vs.* CO-treated $Car^{+/+}$ mice) of hepatic expression for genes involved in cholesterol metabolism (derived from microarray results). C) Fold-change (TCPOBOP- *vs.* CO-treated $Car^{-/-}$ mice) of hepatic expression for genes involved in bile acid metabolism (derived from microarray results). D) Fold-change (TCPOBOP- *vs.* CO-treated $Car^{-/-}$ mice) of hepatic expression for genes involved in cholesterol metabolism (derived from microarray results). *treatment effect. * $p_{adj}<0.05$, ** $p_{adj}<0.01$, *** $p_{adj}<0.001$.

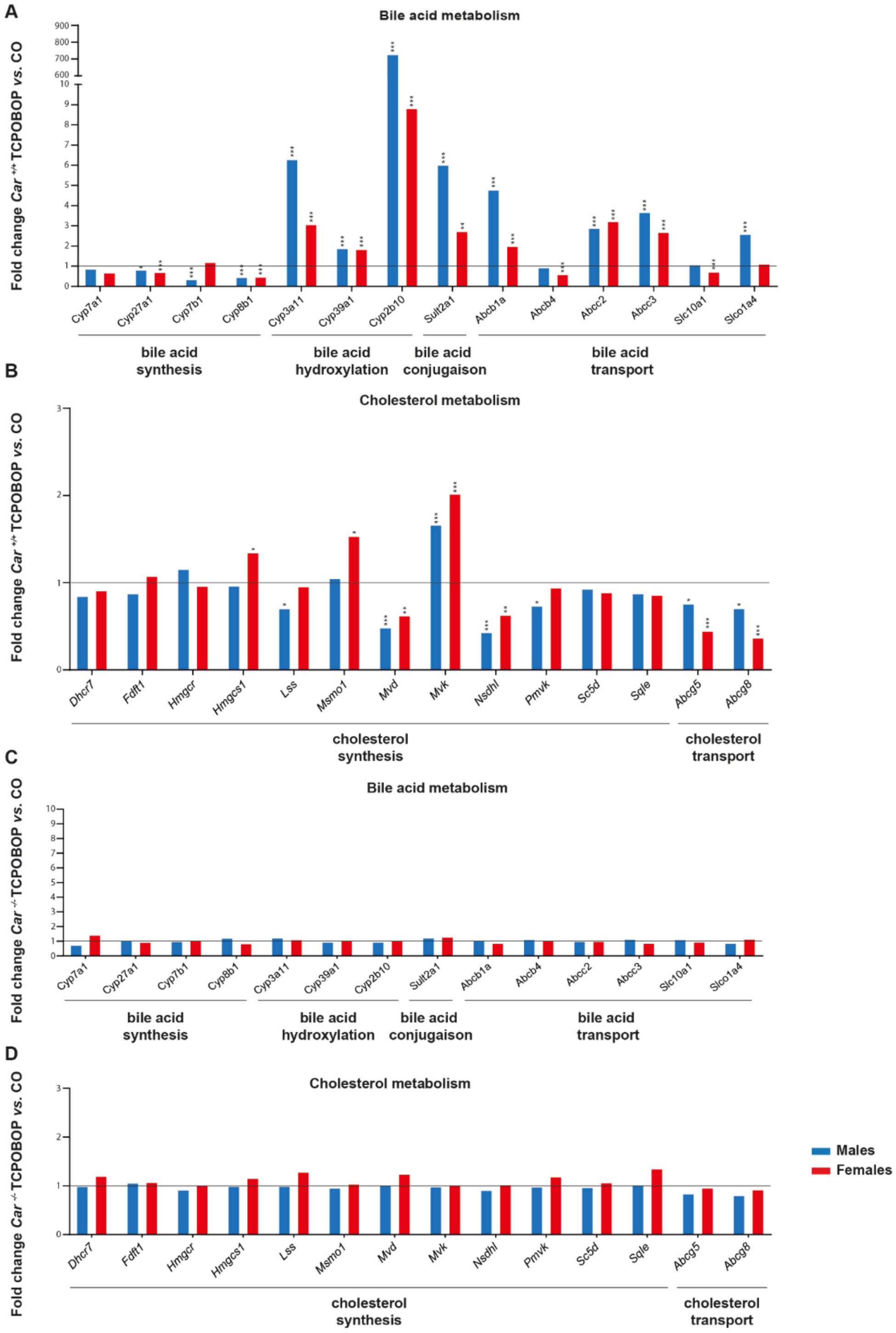

Fig. S7: ¹H-NMR-based metabolomics in liver samples from *Car⁻/⁻* mice. A) Coefficient plots related to the PLS-DA models discriminating between ¹H-NMR-based liver spectra from TCPOBOP *vs.* CO *Car⁻/⁻* males. Parameters of the PLS-DA model: $Q^2Y=-0.18$, $p>0.05$. B) Coefficient plots related to the PLS-DA models discriminating between ¹H-NMR-based liver spectra from TCPOBOP *vs.* CO *Car⁻/⁻* females. Parameters of the PLS-DA model: $Q^2Y=-0.28$, $p>0.05$.

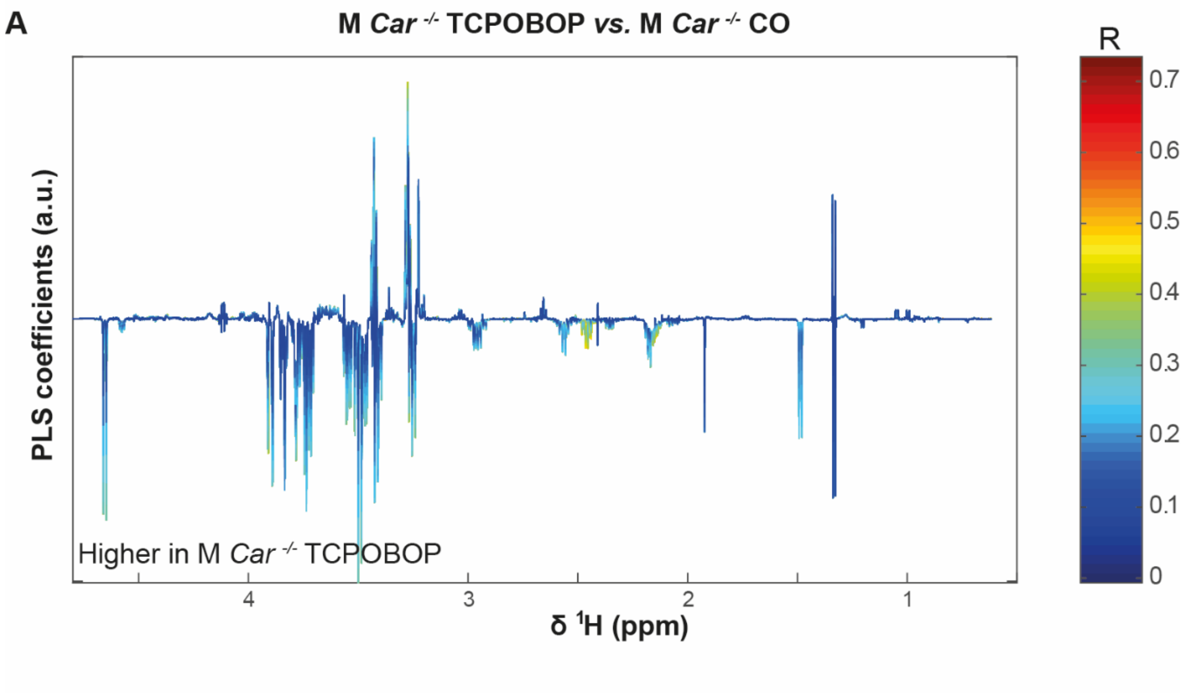

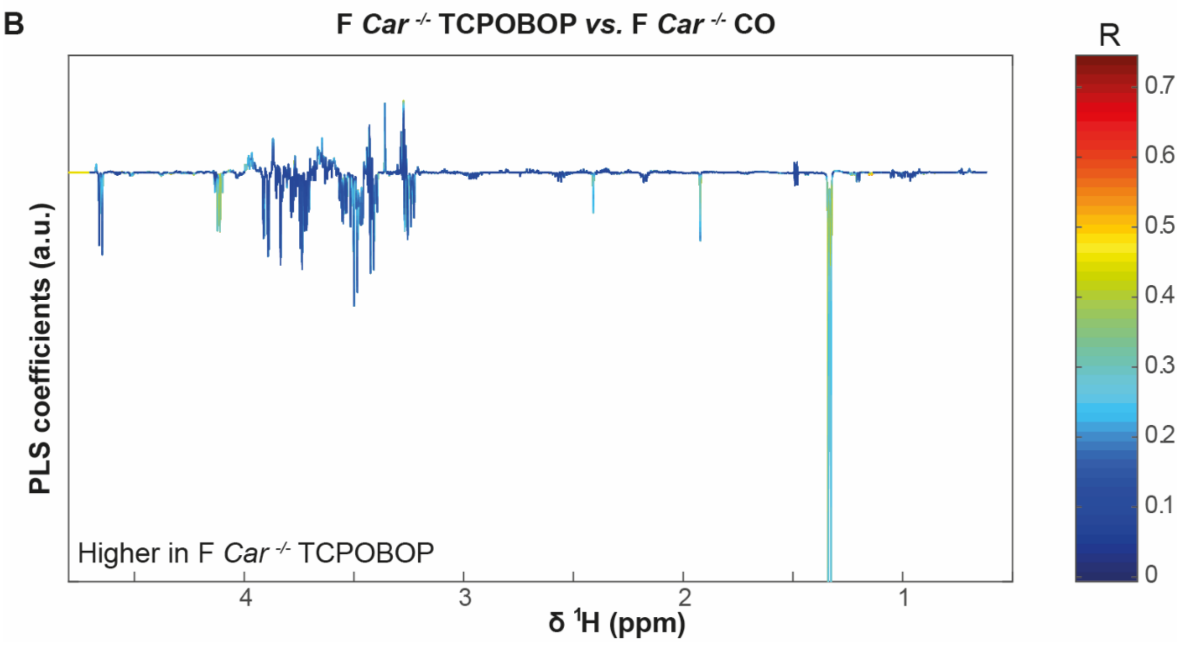

Table S1: Primers used for RT-qPCR.

Gene	NCBI Refseq	Forward primer	Reverse primer
Car	NM_009803	GCTGCAAGGGCTTCTTCAGA	CCTTCCAGCAAACGGACAGA
Cyp2b10	NM_009999	TTTCTGCCCTTCTCAACAGGAA	ATGGACGTGAAGAAAAGGAACAAC
Cyp2c55	NM_028089	TTGTGGAAGAGCTAAGAAAAGCAAAT	GAGCACAGCTCAGGATGAATGT
Fmo3	NM_008030	AAGAAAGGAAGACAAAGAAAAGGCA	AGCTCCAATGATGGCCACTT

Table S2: ^{1}H and partial ^{13}C assignments for identified metabolites in plasma samples. Signals highlighted in bold were used for determination of area under the curves. Keys: s: singlet; d: doublet; t: triplet; q: quadruplet; m: multiplet; dd: doublet of doublet.

x*: tentative assignment

Metabolite	Chemical function	δ ^{1}H (ppm)	Multiplicity	δ ^{13}C (ppm)
Cholesterol (mainly in HDL)		**0.66**	**s (broad)**	
lipid in LDL	$(CH_3(CH_2)_n)$	**0.85**	**s (broad)**	24.6
	(CH2)n	1.23	s (broad)	32.0
lipid in VLDL	$(CH_3CH_3CH_2C=)$	**0.87**	**t**	16.5
	$CH_2CH_2CH_2CO$	1.29	m	
	CH_2CH_2CO	1.59	m	27.0
Leucine		0.95	t	
Valine		0.99	d	
		1.05	d	20.3
Isoleucine		1.01	d	
Ethanol		1.19	t	19.4
		3.65	q	60.4
lactate		**1.33**	**d**	22.4
		4.12	q	71.3
alanine		1.48	d	19.0
unknown		1.73	m	28.3
acetate		1.92	s	26.0
lipid	$CH_2C=C$	2.0	m	29.0
	$CH_2C=C$	2.04	m	
methionine		2.13	s	17.1
lipid	CH_2CO	2.24	t	35.7
3-hydroxybutyrate		2.31	dd	
succinate*		2.4	s	
glutamine		2.45	m	33.5
unknown		2.53	s	34.7
methylamine		2.55	s	24.7
unknown		2.69	s	28.5
dimethylamine*		2.71	s	
lipid		2.76	m	28.3
creatine		3.04	s	42.2
glycerophosphocholine		3.22	s	56.7
β-glucose		3.25	m	77.2
		3.47	m	79.0
		3.9	m	63.5
		4.65	d	98.9
α-glucose		3.41	m	72.5
		3.53	m	72.0
		3.71	m	63.0
		3.78	m	63.7
		3.85	m	74.3

	**5.25**	**d**	95.4
unsaturated lipids	5.3	m	132.2
fumarate*	6.52	s	
tyrosine	6.9	d	118.7
	7.19	d	134.7
unknown	7.05	s	
phenylalanine	7.34	m	146.2
	7.43	t	146.4
unknown	7.75	s	147.0

Table S3: ^{1}H and partial ^{13}C assignments for identified metabolites in liver samples. Signals highlighted in bold were used for determination of area under the curves. Keys: s: singlet; d: doublet; t: triplet; q: quadruplet; m: multiplet; dd: doublet of doublet.

Metabolite	δ ^{1}H (ppm)	Multiplicity	δ ^{13}C (ppm)
Bile acids (mixed)	0.6-0.75	s	
	0.92	s	
Bile acids (tauroconjugated)	0.72	s	
	0.92	s	
	3.08	t	
Leucine	0.96	t	23.6
	1.72	m	
	3.74	m	
Valine	0.99	d	19.5
	1.05	d	20.8
	2.29		
	3.62		
Isoleucine	0.94	t	
	1.01	d	
	1.26	m	
	1.48	m	
	2.0	m	
	3.65	d	
3-hydroxybutyrate	1.19	d	24.4
	2.32	dd	
	2.4	dd	
	4.14	m	
Lactate	1.33	d	22.8
	4.11	q	
Threonine	1.33	d	22.3
	3.61		
	4.23		
Alanine	1.49	d	19.1
	3.79		
Ornithine	1.72	m	
	1.93	m	
	3.03	t	41.9
	3.77	t	
Acetate	1.93	s	
L-glutamate	2.06	m	29.8
	2.35	m	36.4
	3.76	dd	
L-glutamine	2.13	m	29.3
	2.45	m	
	3.77	t	
Glutathion (oxidized)	2.17	t	29.1
	**2.53**	**m**	34.2

	2.98	dd	
	3.31	m	41.6
	3.76	m	
	4.75	m	
Glutathion (reduced)	2.17	m	
	**2.56**	**m**	
	2.95	m	
	3.76	m	
	4.56	dd	
Succinate	2.42	s	36.8
Hypotaurine	**2.65**	**t**	
	3.36	t	
L-aspartic acid	2.66	dd	
	2.8	dd	
	3.89	dd	
Dimethylamine	2.72	s	37.6
Trimethylamine	**2.89**	**s**	
Dimethylglycine	2.93	s	46.5
Creatine	3.04	s	39.9
	3.94	s	56.6
Choline	3.2	s	56.7
	3.55		
O-phosphocholine	3.21	s	56.6
	3.58	m	
	4.13	m	
Betaine	3.27	s	55.9
	3.88	s	
Taurine	3.28	t	50.4
	3.44	t	38.5
Methanol	3.36	s	51.7
β-glucose	3.51	m	
	3.75		
	4.66	d	98.7
AMP	4.01	dd	
	4.36	dd	
	4.5	dd	
	6.14	d	89.6
	8.27	s	
	8.62	s	
α-glucose	3.45	m	
	3.56		
	3.72		
	3.84		
	3.97		
	5.25	d	94.8
Glycine	3.56	s	44.4
UDP-glucose	5.6	dd	
	5.95	d	
	7.93	d	

UDP-glucuronate	5.6	dd	
	5.98	d	
	7.96	d	
Uridine	5.89	d	
	5.9	d	
	7.89	d	
NADP+	6.03	s	
	6.1	d	
	8.15	d	
	8.42	s	
	8.584	s	
	8.82	s	
	9.12	d	
	9.3	s	
NAD+	6.04	d	89.5
	6.09	d	102.6
	8.18	s	
	8.2	dd	
	8.44	s	
	8.84	d	
	9.15	d	
	9.34	s	
Inosine	6.11	d	91.1
	8.23	s	
	8.34	s	
Fumarate	6.52	s	
Tyrosine	6.87	d	
	7.19		132.5
Phenylalanine	3.12	dd	
	3.26	dd	
	7.33	m	
	7.38	m	
	7.43	m	
Nicotinurate	3.99	s	
	7.6	dd	
	8.25	m	
	8.72	dd	
	8.94	s	
Nicotinamide	7.61	dd	
	8.23	dd	
	8.7	dd	
	8.95	s	

Table S4: Fold-changes, p-values and pathway enrichment analysis for all TCPOBOP modulated genes (related to Fig. 2c). A) List of genes contained in cluster 1 with their Log(fold-change) values and adjusted p-values in *Car*$^{+/+}$ males and females. B) Gene Ontology (GO) term enrichment analysis using the list of genes contained in cluster 1. C) List of genes contained in cluster 2 with their Log(fold-change) values and adjusted p-values in males and females *Car*$^{+/+}$. D) GO term enrichment analysis using the list of genes contained in cluster 2. Metabolic pathways were considered as significantly enriched when $P_{adj}<0.05$.

Table S5: Expression of previously-described CAR target genes in both sexes (related to Fig. 2e-g). Table presenting list of genes well-described in the literature as CAR-sensitive genes with their Log(fold-change) values and adjusted p-values in males and females *Car*$^{+/+}$. Data were derived from liver microarray data.

Table S6: Fold-changes, p-values and pathway enrichment analysis for genes modulated by TCPOBOP in a sex-dependent manner (related to Fig. 3b). A) Genes contained in cluster 1 with their Log(fold-change) values and adjusted p-values in *Car*$^{+/+}$ males and females. B) GO term enrichment analysis. C) Genes contained in cluster 2 with their Log(fold-change) values and adjusted p-values in males and females *Car*$^{+/+}$ groups. D) GO term enrichment analysis. E) List of genes contained in cluster 3 with their Log(fold-change) values and adjusted p-values in males and females *Car*$^{+/+}$ groups. F) GO term enrichment analysis. G) List of genes contained in cluster 4 with their Log(fold-change) values and adjusted p-values in males and females *Car*$^{+/+}$ groups. H) GO term enrichment analysis.

Supplementary references

1. Rox, K., Rath, S., Pieper, D. H., Vital, M. & Brönstrup, M. A simplified LC-MS/MS method for the quantification of the cardiovascular disease biomarker trimethylamine-N-oxide and its precursors. *J. Pharm. Anal.* 11, 523–528 (2021).
