# Supplementary material for: Pharmacological activation of constitutive androstane receptor induces female-specific modulation of hepatic metabolism
Source: JHEP Rep. 2023 Oct 13;6(1):100930. doi: 10.1016/j.jhepr.2023.100930 (PMC10749885; doi:10.1016/j.jhepr.2023.100930)
Supplement: Multimedia component 5 [file mmc5.docx]

**Journal of Hepatology**

**CTAT methods**

Tables for a “Complete, Transparent, Accurate and Timely account” (CTAT) are now mandatory for all revised submissions. The aim is to enhance the reproducibility of methods.

- Only include the parts relevant to your study
- Refer to the CTAT in the main text as ‘Supplementary CTAT Table’
- Do not add subheadings
- Add as many rows as needed to include all information
- Only include one item per row

**If the CTAT form is not relevant to your study, please outline the reasons why:**

|  |
| --- |

- 1. **Antibodies**

| **Name** | **Citation** | **Supplier** | **Cat no.** | **Clone no.** |
| --- | --- | --- | --- | --- |
|  |  |  |  |  |

- 1. **Cell lines**

| **Name** | **Citation** | **Supplier** | **Cat no.** | **Passage no.** | **Authentication test method** |
| --- | --- | --- | --- | --- | --- |
|  |  |  |  |  |  |

- 1. **Organisms**

| **Name** | **Strain** | **Sex** | **Age** | **Overall n number** |
| --- | --- | --- | --- | --- |
| Mus musculus | C57BL6/J | Males and females | 9-week-old | 108 |

- 1. **Sequence based reagents**

| **Name** | **Sequence** | **Supplier** |
| --- | --- | --- |
| Agilent Sureprint G3 Mouse GE v2 microarrays | design 074809 | Agilent |

- 1. **Biological samples**

| **Description** | **Source** | **Identifier** |
| --- | --- | --- |
|  |  |  |

- 1. **Deposited data**

| **Name of repository** | **Identifier** | **Link** |
| --- | --- | --- |
| NCBI Gene Expression Omnibus | GSE228554 | https://www.ncbi.nlm.nih.gov/geo/query/acc.cgi?acc=GSE228554 |

- 1. **Software**

| **Software name** | **Manufacturer** | **Version** |
| --- | --- | --- |
| R | free | 4.1.0 |
| Matlab | The MathWorks, Inc | 2021a |
| GraphPad Prism | GraphPad Software LLC | 10.0.1 |

- 1. **Other (e.g. drugs, proteins, vectors etc.)**

|  |  |  |
| --- | --- | --- |
|  |  |  |

- 1. **Please provide the details of the corresponding methods author for the manuscript:**

| Dr Ellero-Simatos Sandrine; Toxalim (Research Centre in Food Toxicology); INRAE, ENVT, INP-Purpan, UPS, Université de Toulouse ; 180 chemin de Tournefeuille,; BP.93173; 31027 TOULOUSE Cedex 3, France  E-Mail : sandrine.ellero-simatos@inrae.fr |
| --- |

**2.0 Please confirm for randomised controlled trials all versions of the clinical protocol are included in the submission. These will be published online as supplementary information.**

|  |
| --- |
