# Supplementary material for: Pharmacological activation of constitutive androstane receptor induces female-specific modulation of hepatic metabolism
Source: JHEP Rep. 2023 Oct 13;6(1):100930. doi: 10.1016/j.jhepr.2023.100930 (PMC10749885; doi:10.1016/j.jhepr.2023.100930)
Supplement: Multimedia component 7 [file mmc7.pdf]

Research article

JHEP|Reports

Pharmacological activation of constitutive androstane receptor induces female-specific modulation of hepatic metabolism

Authors

Marine Huillet, Frédéric Lasserre, Marie-Pierre Gratacap, Beatrice Engelmann, Justine Bruse, Arnaud Polizzi, Tiffany Fougeray, Céline Martin, Clémence Rives, Anne Fougerat, Claire Naylies, Yannick Lippi, Géraldine Garcia, Elodie Rousseau-Bacquie, Cécile Canlet, Laurent Debrauwer, Ulrike Rolle-Kampczyk, Martin von Bergen, Bernard Payrastre, Elisa Boutet-Robinet, Laurence Gamet-Payrastre, Hervé Guillou, Nicolas Loiseau, Sandrine Ellero-Simatos

Correspondence

sandrine.ellero-simatos@inrae.fr (S. Ellero-Simatos).

Graphical abstract

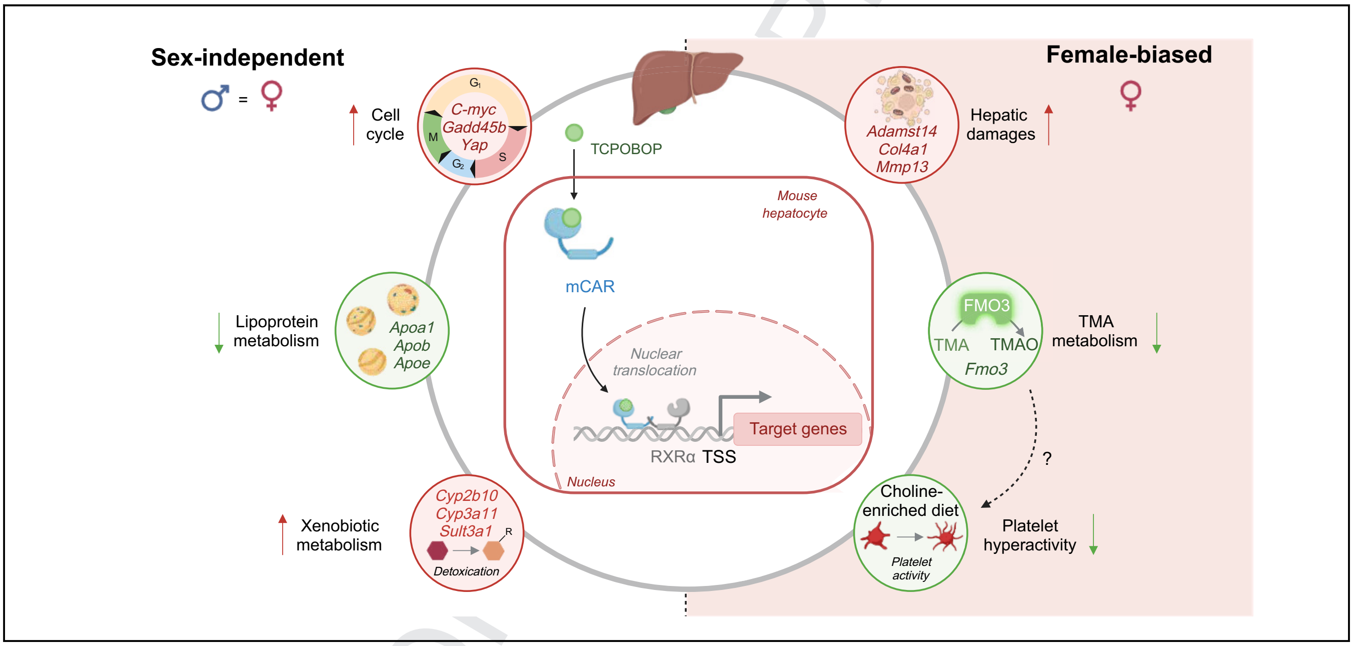

Highlights

- Sex-independent *vs.* sex-biased CAR-dependent hepatic pathways were defined.
- CAR activation influenced circulating lipoproteins in a sex-independent manner.
- CAR activation influenced liver metabolism in female mice more than in males.
- CAR activation inhibited TMAO synthesis, which could influence platelet aggregation.

Impact and implications

CAR is activated by many drugs and pollutants. Its pharmacological activation had a stronger impact on hepatic gene expression and metabolism in females than in males, and had a specific impact on liver toxicity and trimethylamine metabolism. Sexual dimorphism should be considered when testing and/ or prescribing xenobiotics known to activate CAR.

https://doi.org/10.1016/j.jhepr.2023.100930

UNCORRECTED PROOF

Pharmacological activation of constitutive androstane receptor induces female-specific modulation of hepatic metabolism

Marine Huillet,[1] Frédéric Lasserre,[1] Marie-Pierre Gratacap,[2] Beatrice Engelmann,[3] Justine Bruse,[1] Arnaud Polizzi,[1] Tiffany Fougeray,[1] Céline Marie Pauline Martin,[1] Clémence Rives,[1] Anne Fougerat,[1] Claire Naylies,[1] Yannick Lippi,[1] Géraldine Garcia,[1] Elodie Rousseau-Bacquie,[1] Cécile Canlet,[1] Laurent Debrauwer,[1] Ulrike Rolle-Kampczyk,[3] Martin von Bergen,[3] Bernard Payrastre,[2,4] Elisa Boutet-Robinet,[1] Laurence Gamet-Payrastre,[1] Hervé Guillou,[1] Nicolas Loiseau,[1] Sandrine Ellero-Simatos[1,*]

[1]Toxalim (Research Centre in Food Toxicology), INRAE, ENVT, INP-Purpan, UPS, Université de Toulouse, Toulouse, France; [2]INSERM, UMR-1297 and Université Toulouse III, Institut de Maladies Métaboliques et Cardiovasculaires (I2MC), CHU-Rangueil, Toulouse, France; [3]Department of Molecular Systems Biology, Helmholtz Centre for Environmental Research, Leipzig, Germany; [4]Laboratoire d'Hématologie, CHU de Toulouse, Toulouse, France

JHEP Reports **2024.** https://doi.org/10.1016/j.jhepr.2023.100930

Background & Aims: The constitutive androstane receptor (CAR) is a nuclear receptor that binds diverse xenobiotics and whose activation leads to the modulation of the expression of target genes involved in xenobiotic detoxification and energy metabolism. Although CAR hepatic activity is considered to be higher in women than in men, its sex-dependent response to an acute pharmacological activation has seldom been investigated.
Methods: The hepatic transcriptome, plasma markers, and hepatic metabolome, were analysed in *Car*$^{+/+}$ and *Car*$^{-/-}$ male and female mice treated either with the CAR-specific agonist 1,4-bis[2-(3,5-dichloropyridyloxy)]benzene (TCPOBOP) or with vehicle.
Results: Although 90% of TCPOBOP-sensitive genes were modulated in a sex-independent manner, the remaining 10% showed almost exclusive female liver specificity. These female-specific *CAR*-sensitive genes were mainly involved in xenobiotic metabolism, inflammation, and extracellular matrix organisation. CAR activation also induced higher hepatic oxidative stress and hepatocyte cytolysis in females than in males. Hepatic expression of flavin monooxygenase 3 (*Fmo3*) was almost abolished and was associated with a decrease in hepatic trimethylamine-N-oxide (TMAO) concentration in TCPOBOP-treated females. In line with a potential role in the control of TMAO homeostasis, CAR activation decreased platelet hyper-responsiveness in female mice supplemented with dietary choline.
Conclusions: More than 10% of CAR-sensitive genes are sex-specific and influence hepatic and systemic responses such as platelet aggregation. CAR activation may be an important mechanism of sexually-dimorphic drug-induced liver injury.
Impact and implications: CAR is activated by many drugs and pollutants. Its pharmacological activation had a stronger impact on hepatic gene expression and metabolism in females than in males, and had a specific impact on liver toxicity and trimethylamine metabolism. Sexual dimorphism should be considered when testing and/or prescribing xenobiotics known to activate CAR.
© 2023 The Authors. Published by Elsevier B.V. on behalf of European Association for the Study of the Liver (EASL). This is an open access article under the CC BY license (http://creativecommons.org/licenses/by/4.0/).

Introduction

The liver is a highly sexually dimorphic organ. There is increasing evidence for sexually-dimorphic regulation of xenobiotic clearance, responses to drugs and drug-induced liver injury. We postulated that the mechanisms underlying such dimorphism may involve the constitutive androstane receptor (CAR, NR1I3). CAR is a liver-enriched member of the nuclear receptor superfamily that controls ligand-dependent regulation of gene expression. Upon ligand-binding, CAR translocates to the nucleus, heterodimerises with retinoid X receptor α, and binds the xenobiotic response element located on DNA, upstream of the promoter sequences of its target genes. CAR was first described as a xenobiotic receptor that recognises a wide variety of drugs, foods, and environmental pollutants.[1,2] Later studies then unveiled that CAR can also be activated by endobiotics such as bilirubin, bile acids, and steroid hormones.[3,4] Upon activation, CAR regulates the expression of critical enzymes involved in phase I, II, and III xenobiotic metabolism pathways,[5,6] thereby playing a central role in xenobiotic detoxification and clearance. Moreover, CAR is involved in glucose and lipid homeostasis, although its exact role in hepatic metabolism remains controversial.[7]

Keywords: Sexual dimorphism; Hepatic xenobiotic metabolism; Lipoprotein metabolism; Platelet aggregation; Trimethylamine-N-oxide.
Received 17 April 2023; received in revised form 25 September 2023; accepted 27 September 2023; available online 13 October 2023
* Corresponding author. Address: Toxalim (Research Centre in Food Toxicology); INRAE, ENVT, INP-Purpan, UPS, Université de Toulouse, 180 chemin de Tournefeuille, BP.93173, 31027 Toulouse Cedex 3, France. Tel: +33 582066349.
E-mail address: sandrine.ellero-simatos@inrae.fr (S. Ellero-Simatos).

ELSEVIER
EASL The Home of Hepatology

Hepatic expression of *Car* and its main target genes *Cyp2b9* and *Cyp2b10* is higher in female mice than in males.[8,9] Moreover, treatment with nonylphenol, a moderate CAR activator, induced expression of cytochromes P450 (CYPs) more strongly in the female mouse liver than in males.[10] Similarly, treatment with 1,4-bis[2-(3,5-dichloropyridyloxy)]benzene (TCPOBOP), the prototypical pharmacological agonist of murine CAR (mCar),[11] increased liver proliferation in female more than in male mice.[12] Our previous study showed that deletion of CAR expression had a stronger impact on female hepatic gene expression than on males; however, CAR-deleted males developed spontaneous steatosis during ageing while females did not.[13] Therefore, CAR is thought to impact rodent liver gene expression in a sex-dependent manner, with higher CAR activity and higher sensitivity to CAR activation in females. Interestingly, in humans, the expression and activity of CYP2B6, the prototypical target gene for human CAR (hCAR), were higher in the liver of women compared with men, indicating that sexual dimorphism of CAR activity was also transposable to humans.[8] Despite the recognition of its sexually dimorphic activity, *in vivo* studies conducted so far on both male and female mice have focused on the impact of CAR activation on the regulation of CYPs,[14] on genes involved in cell cycle and hepatocarcinogenesis,[15,16] or on long non-coding RNA.[17] A genome-wide comparison of the effects of CAR activation in male and female mice is still lacking.

In this study, we used hepatic microarray and metabolomics analysis of wild-type (*Car*$^{+/+}$) and whole-body knockout littermate (*Car*$^{-/-}$) male and female mice treated with either Corn oil (CO, vehicle) or TCPOBOP to elucidate the potential sex-dependent impact of CAR activation.

Materials and methods

Animal models

In vivo studies were performed in a conventional laboratory animal room following the European Union Guidelines for Use and Care of Laboratory Animals. This project was approved by an independent ethics committee (CEEA-86 Toxcométhique, authorisation number 2019123014045837). The animals were treated humanely with due consideration to the alleviation of distress and discomfort. All mice were housed at 22 °C ± 2 °C on a 12-h light (ZT0–ZT12) 12-h dark (ZT12–ZT24) cycle, where ZT indicates Zeitgeber time; ZT0 is defined as the time when the lights are turned on. Animals were allowed free access to food (Teklad Global 18% Protein Rodent Diet) and tap water. *Car*$^{-/-}$ mice (backcrossed on the C57BL/6J) were engineered by Wei *et al.*[1] and were bred for 15 years in our animal facility. *Car*$^{+/-}$ mice were co-bred and gave birth to true *Car*$^{+/+}$ and *Car*$^{-/-}$ littermate mice, which were then separated by sex and genotype at 4 weeks of age and were randomly allocated to the different experimental groups. Nine-week-old male and female mice included in TCPOBOP groups received a daily intraperitoneal injection of 1,4-bis[2-(3,5-dichloropyridyloxy)]benzene (TCPOBOP, Sigma Aldrich) diluted in CO used as vehicle at 3 mg/kg for 4 days, whereas CO mice received CO only (Sigma Aldrich). One cage housing n = 6 mice per group was used. At ZT16 (6 h after the last TCPOBOP injection), mice were anaesthetised with isoflurane and xylazine (2%, 2 mg/kg) then blood from the vena cava was collected into lithium heparin-coated tubes (BD Microtainer, Franklin Lake, NJ, USA). Plasma was prepared by centrifugation (1,500×*g*, 15 min, 4 °C) and stored at −80 °C. Following euthanasia by cervical dislocation, the liver and perigonadal white adipose tissue were removed, weighted and snap-frozen in liquid nitrogen, and then stored at −80 °C until use.

To confirm *Fmo3* regulation upon TCPOBOP treatment an independent experiment was conducted using the same experimental groups but with different timing of TCPOBOP treatment leading to the same total dose of TCPOBOP: intraperitoneal injection either with TCPOBOP diluted in CO at 3 mg/kg every 2 days for 10 days or with CO, at ZT10. Mice were euthanised by cervical dislocation at ZT8 and liver was removed, weighed and snap-frozen in liquid nitrogen and stored at −80 °C.

For thrombus formation analysis, another set of 7-week-old C57BL/6J mice were purchased from Charles River laboratories, acclimatised for 2 weeks, then randomly allocated to the different experimental groups: Female Corn Oil (F CO, n = 10), female TCPOBOP (F TCPOBOP, n = 10), male Corn Oil (M CO, n = 10), male TCPOBOP (M TCPOBOP, n = 10) (two cages per group). Then, mice were fed with 1% choline-enriched diet (D13090101, Research Diets) for 10 days. Mice included in the TCPOBOP groups received an intraperitoneal injection of TCPOBOP diluted in CO at 3 mg/kg for the last 4 days of diet, whereas CO mice received CO only, at ZT0. Between ZT3 and ZT8 whole blood was drawn from the inferior vena cava of anaesthetised mice (100 mg/kg ketamine, 10 mg/kg xylazine) into heparin sodium (10 IU/ml) and mice were euthanised by cervical dislocation and liver was removed, weighed and snap-frozen in liquid nitrogen and stored at −80 °C until use.

Gene expression

Gene expression profiles were performed at the GeT-TRiX facility (GénoToul, Génopole Toulouse Midi-Pyrénées, Toulouse, France) using Agilent Sureprint G3 Mouse GE v2 microarrays (8 × 60K, design 074809) following the manufacturer's instructions. Data acquisition and statistical analyses were performed as previously described.[18] A correction for multiple testing was applied using the Benjamini-Hochberg procedure to control the false discovery rate (FDR). Probes with fold change (FC) ≥1.5 and FDR ≤0.05 were considered to be differentially expressed between conditions. The enrichment of Gene Ontology (GO) biological processes was evaluated using Metascape.[19] Data are available in NCBI's Gene Expression Omnibus and are accessible through GEO Series accession number GSE228554.

For real-time quantitative polymerase chain reaction (RT-qPCR), 2 μg RNA samples were reverse-transcribed using the High-Capacity cDNA Reverse Transcription Kit (Applied Biosystems, Foster City, CA, USA). Table S1 presents the SYBR Green assay primers. Amplifications were performed using an ABI Prism 7300 Real-Time PCR System (Applied Biosystems). RT-qPCR data were normalised to TATA-box-binding protein (*Tbp*) mRNA levels.

Proton nuclear magnetic resonance (^{1}H-NMR)-based metabolomics

Plasma samples and liver polar extracts were prepared and analysed using ^{1}H-NMR-based metabolomics. All spectra were obtained on a Bruker DRX-600-Avance NMR spectrometer (Bruker) on the AXIOM metabolomics platform (MetaToul). Details on experimental procedures, data pre-treatment and statistical analysis were described previously.[18] Parameters of the final discriminating orthogonal projection on latent structure-discriminant analysis (O-PLS-DA) are indicated in the figure legends. To identify metabolites responsible for discrimination

between the groups, the O-PLS-DA correlation coefficients (r^2) were calculated for each variable. Correlation coefficients above the threshold defined by Pearson's critical correlation coefficient (p <0.05; |r| >0.7; for n = 6 per group) were considered significant. For illustration purposes, the area under the curve of several signals of interest was integrated and significance tested with two-way ANOVA as described below. For metabolite identification $^{1}H-^{13}C$ heteronuclear single quantum coherence (HSQC) spectra were obtained on one representative sample for each biological matrix. Lists of metabolites measured are presented in Table S2 and S3.

Multi-omics analyses

Bidirectional correlations between plasma metabolites and hepatic transcripts were investigated using N-integration discriminant analysis with DIABLO, an algorithm that aims to identify a highly correlated multi-omics signature discriminating several experimental groups using the R package Mixomics v6.10.9.[20] We used two components in the models, and for the estimation of model parameters, the cross-validation procedure method was used. For the correlation networks, only correlations with a Spearman's rank correlation coefficient >0.96 were plotted.

Analyses of plasma markers

Alanine aminotransferase (ALT), phosphatase alkaline (ALP), total cholesterol, high-density lipoprotein (HDL-cholesterol), triglycerides, and free fatty acids were determined using an ABX Pentra 400 biochemical analyser (Anexplo facility, Toulouse, France). Blood glucose levels were measured from the vena cava with an AccuCheck Performa glucometer (Roche Diagnostics).

Trimethylamine-N-oxide targeted LC-MS/MS measurement

For trimethylamine-N-oxide (TMAO) extraction and measurement, see details in the supplementary methods.

Publicly available datasets and databases

Four independent gene expression datasets were found on the Gene Expression Omnibus data repository accessed in September 2019. GSE149229 compared hepatic transcriptome of humanised CAR mice (hCAR) fed a control diet or a phenobarbital (PB)-enriched diet. GSE98666 compared hepatic transcriptome of hCAR mice treated with CO, TCPOBOP or 6-(4-chlorophenyl) imidazo[2,1-b][1,3]thiazole-5-carbaldehyde-O-(3,4-dichlorobenzyl)oxime (CITCO). GSE149228 and GSE57056 compared the hepatic transcriptome of chimeric mice with most human hepatocytes fed a control diet or a PB-enriched diet. Values for *Fmo3* gene expression were calculated using the GEO2R tool for microarray data and using GREIN[21] for RNA sequencing data.

Thrombi formation under flow

Biochips microcapillaries (Vena8Fluoro+, Cellix) were coated with a collagen fibril suspension (50 µg/ml) and saturated with a solution of 0.5% bovine serum albumin in phosphate-buffered saline without Ca^{2+}/Mg^{2+}. Mouse blood was transferred into heparin (10 IU/ml), and DIOC6 (2 µM) was used to label platelets in whole blood. Using a syringe pump (PHD-2000; Harvard Apparatus) to apply a negative pressure, labelled blood was then perfused through a microcapillary for indicated time at a wall shear rate of 1,500 s^{-1} (67.5 dynes/cm^2). Thrombus formation was visualised with an ×40 oil immersion objective for both fluorescent and transmitted light microscopy; the light source was provided by Colibri (Zeiss) and was recorded in real-time (one frame every 20 s). Thrombi volumes were calculated by thresholding of the surface covered by thrombi on a slice of Z-stack images using IMARIS software.[22]

Other statistical analyses

All univariate statistical analyses were performed using GraphPad Prism v.9 (GraphPad Software, San Diego, CA, USA). Outliers were identified using the ROUT method. The Kolmogorov–Smirnov test of normality was applied to all data. Two-way ANOVA was performed within each genotype using sex (male or female) and treatment (CO or TCPOBOP) as fitting factors for the models and a p value representing interactions were reported. If $p_{sex*treatment}$ was significant, Sidak's multiple comparisons test was used as a *post-hoc* test to determine which group differed from its appropriate control, otherwise p values representing the main effects from the ANOVA model (namely sex or treatment) were reported. For platelet aggregation measures, a mixed-effect model was fitted using time and treatment as fixed effects. A p <0.05 was considered significant. Results are given as the mean ± SEM.

Results

Analysis of hepatic transcripts revealed a majority of sex-independent CAR-target genes upon TCPOBOP treatment

To investigate the potential sex-dependent consequences of CAR activation, we treated *Car*$^{+/+}$ and *Car*$^{-/-}$ male and female mice with TCPOBOP (Fig. 1A). TCPOBOP did not affect the total body, decreased perigonadal white adipose tissue and increased liver weights in *Car*$^{+/+}$ male and female mice (Fig. 1B). We confirmed *Car* deletion and observed increased expression of *Cyp2b10* and *Cyp2c55*, two prototypical CAR target genes, in TCPOBOP-treated *Car*$^{+/+}$ males and females. *Cyp2c55* induction by TCPOBOP was significantly higher in females (Fig. 1C). We characterised the impact of CAR activation by TCPOBOP on hepatic gene expression using microarrays. TCPOBOP was a very specific CAR agonist since there was no significantly regulated gene in *Car*$^{-/-}$ male mice, and only two in *Car*$^{-/-}$ female mice (Fig. S1), we thus continued our analysis using *Car*$^{+/+}$ mice only. Principal component analysis (PCA) of the entire expression data set from *Car*$^{+/+}$ mice revealed that individuals clustered separately according to treatment on the first axis and to sex on the second axis (Fig. 1D), illustrating a major effect of CAR activation on the liver transcripts. Comparison of the lists of differentially expressed genes (DEGs) upon TCPOBOP treatment in males *vs.* females *Car*$^{+/+}$ demonstrated that more than half of TCPOBOP-modulated genes were common to males and females (Fig. 1E and Fig. S2). Using all 4663 TCPOBOP-sensitive genes, we highlighted two gene clusters that exhibited sex-independent responses (Fig. 1F). Genes upregulated upon TCPOBOP (cluster 1, 2,366 genes) were mainly involved in the 'cell cycle' (p = 10^{-46}) and 'cellular response to xenobiotic stimulus' (p = 10^{-25}, Fig. 1F and Table S4B), whereas downregulated genes (cluster 2, 2297 genes) were enriched for 'carboxylic acid catabolic process' (p = 10^{-17}) and 'regulation of lipid metabolic process' (p = 10^{-14}, Fig. 1F and Table S4D). We focused on well-described CAR target genes involved in the cell cycle (Fig. 1G) and carbohydrate and lipid metabolism (Fig. 1H) and confirmed that regulation upon TCPOBOP treatment was similar in both males and females (Table S5).

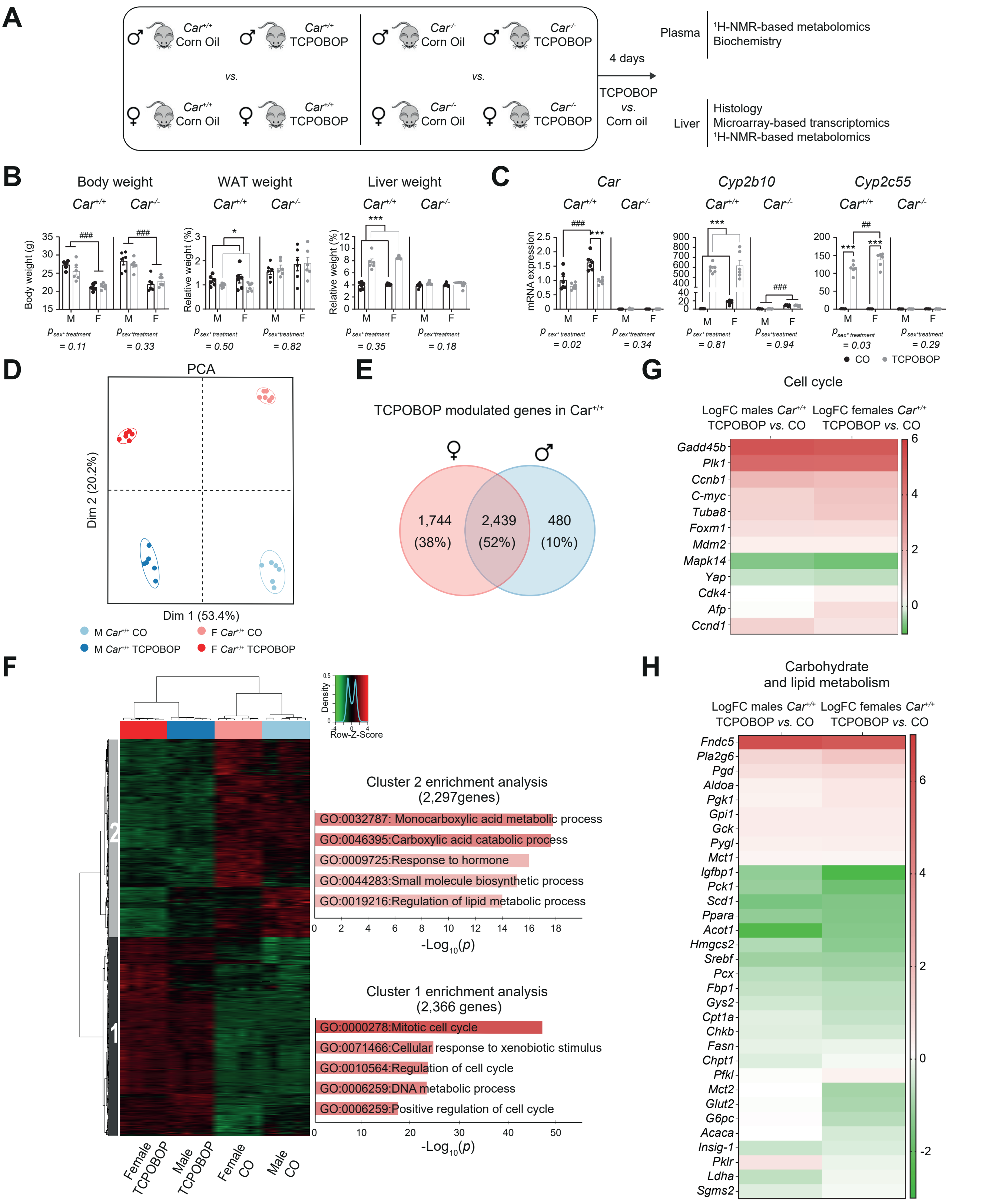

Fig. 1. Modulation of classical CAR-controlled pathways is sex-independent. (A) Experimental design. (B) Body weight, perigonadal WAT, and liver weights. (C) Hepatic gene expression. Results are given as the mean ± SEM. *Treatment effect, #sex effect. * or #p <0.05, ** or ##p <0.01, *** or ###p <0.001 (two-way ANOVA). (D) PCA of the whole liver transcriptomic dataset in *Car*$^{+/+}$ mice. (E) Venn diagram representing the number of genes significantly modulated by TCPOBOP in the liver of *Car*$^{+/+}$ male and female mice (p_{adj} <0.05 and fold-change >1.5). (F) Hierarchical clustering and pathway enrichment analysis using the 4,663 genes significantly regulated upon TCPOBOP in males or females (p_{adj} <0.05 and fold-change >1.5). (G) Heatmaps representing the log(fold-change) of gene expression between TCPOBOP- and CO-treated *Car*$^{+/+}$ males and females and (H) for genes involved in cell cycle. and carbohydrate and lipid metabolism. *CAR*, constitutive androstane receptor; CO, corn oil; PCA, principal component analysis; TCPOBOP, 1,4-bis[2-(3,5-dichloropyridyloxy)] benzene; WAT, white adipose tissue.

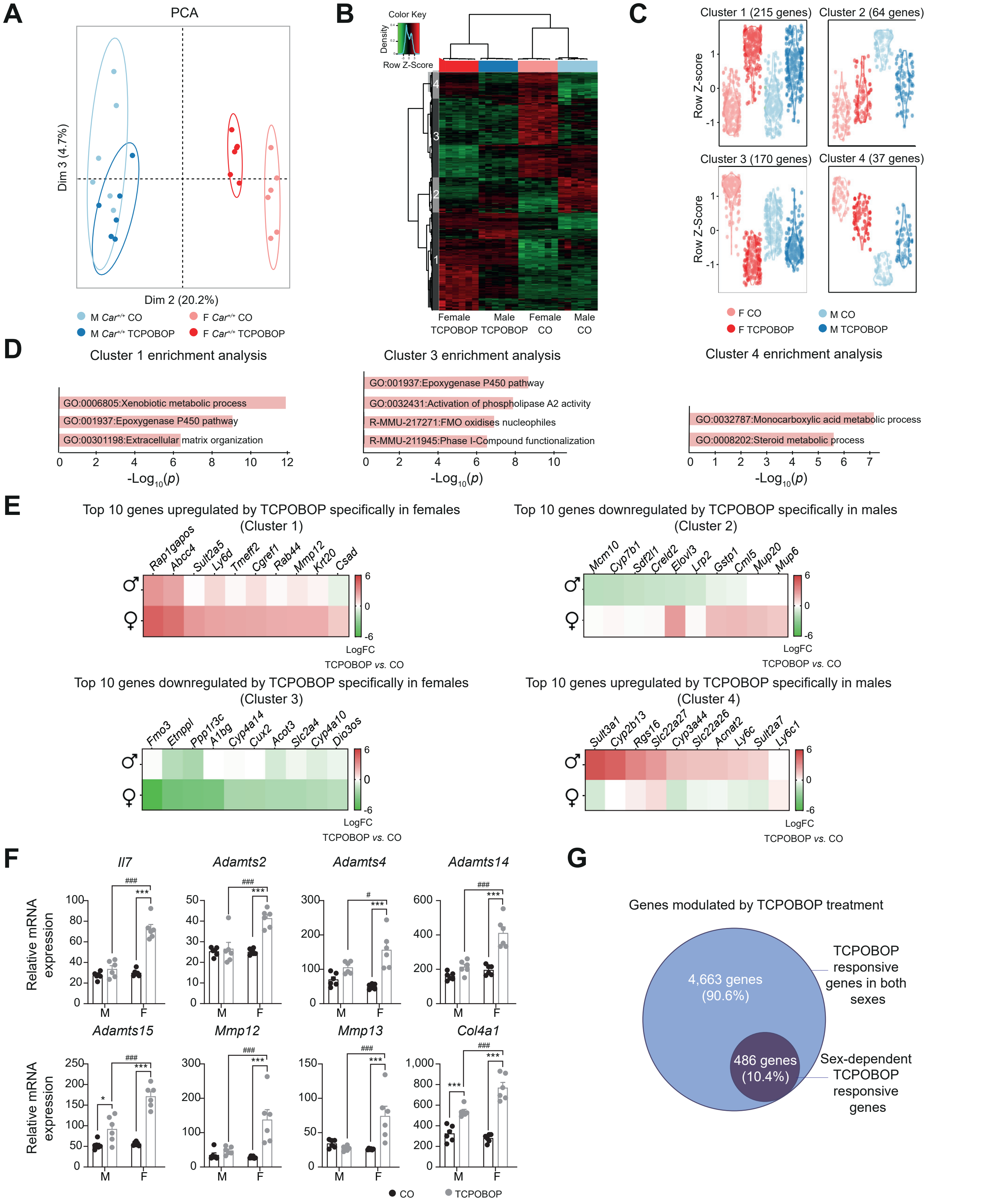

Fig. 2. Identification of sex-dependent CAR-sensitive genes. (A) PCA of the liver transcriptomic dataset in *Car*$^{+/+}$ *mice.* (B) Hierarchical clustering using the 486 genes with significant $p_{sex*treatment}$ (p_{adj} <0.05 and fold-change>1.5). (C) Gene expression profiles in each cluster. (D) Pathway enrichment analysis. (E) Heatmaps representing the log(fold-change) of gene expression between TCPOBOP- and CO-treated *Car*$^{+/+}$ males and females for the top 10 genes in each cluster. (F) Hepatic gene expression of genes involved in inflammation and fibrosis derived from microarray data. *Treatment effect, #sex effect. * *or* # p_{adj} <0.05, ** *or* ## p_{adj} <0.01, *** *or* ### p_{adj} <0.001 (linear models). (G) Genes modulated by TCPOBOP treatment in a sex-dependent and -independent way. CAR, constitutive androstane receptor; CO, corn oil; PCA, principal component analysis; TCPOBOP, 1,4-bis[2-(3,5-dichloropyridyloxy)] benzene.

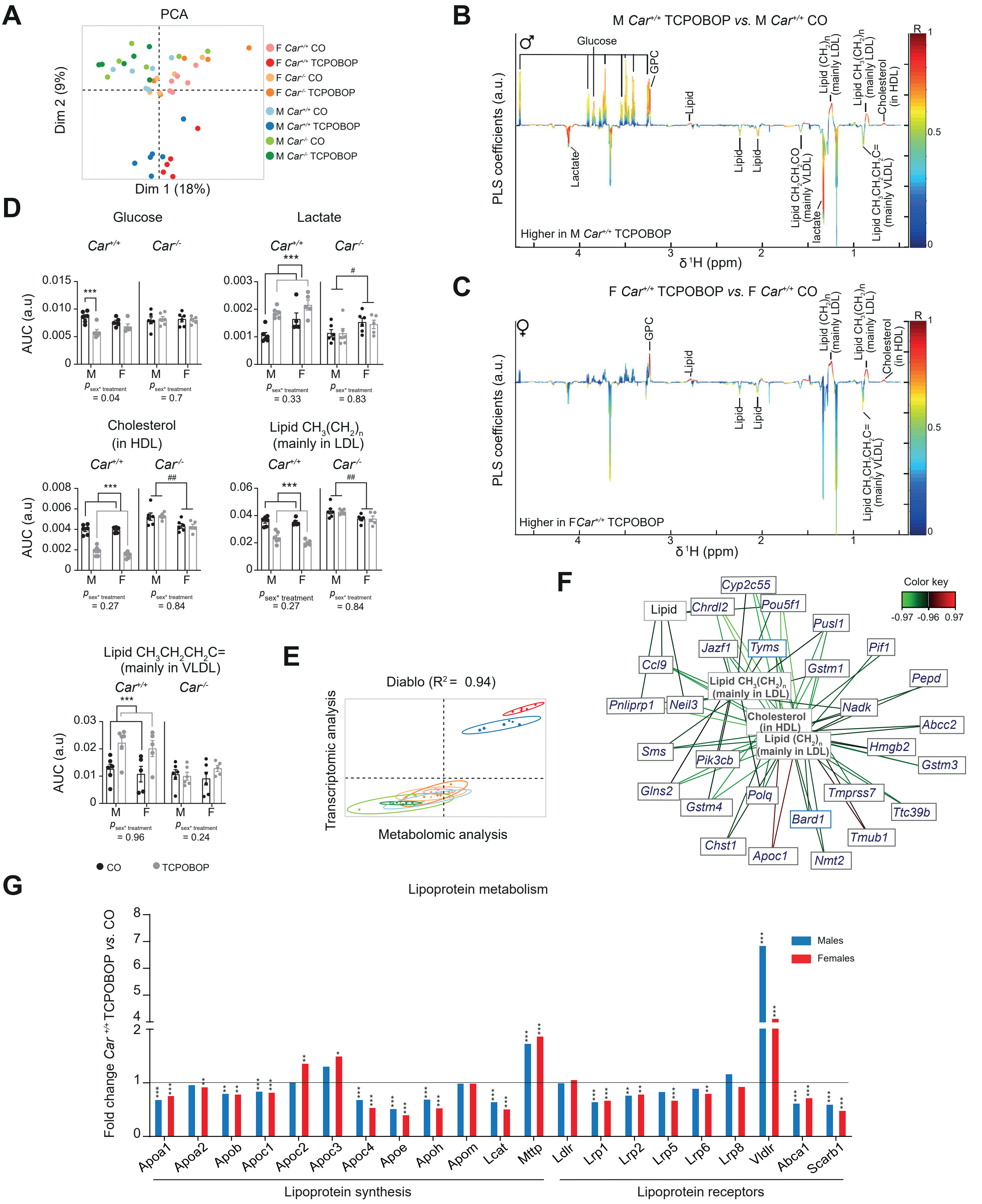

Fig. 3. Sex-independent impact of CAR activation on lipoprotein metabolism. (A) PCA of plasma metabolomic dataset. (B) Coefficient plots related to the PLS-DA models discriminating between plasma spectra from TCPOBOP *vs.* CO $Car^{+/+}$ males. Parameters of the PLS-DA model: $Q^2Y = 0.84$, $p = 0.001$. (C) Coefficient plots related to the PLS-DA models discriminating between plasma spectra from TCPOBOP *vs.* CO $Car^{+/+}$ females. Parameters of the PLS-DA model: $Q^2Y = 0.89$, $p = 0.001$. (D) AUC of the ^{1}H-NMR spectra was integrated for glucose, lactate, cholesterol, lipid $CH_3(CH_2)_n$ (mainly in Ldl), lipid $CH_3CH_2CH_2C$ = (mainly in Vldl) signals.

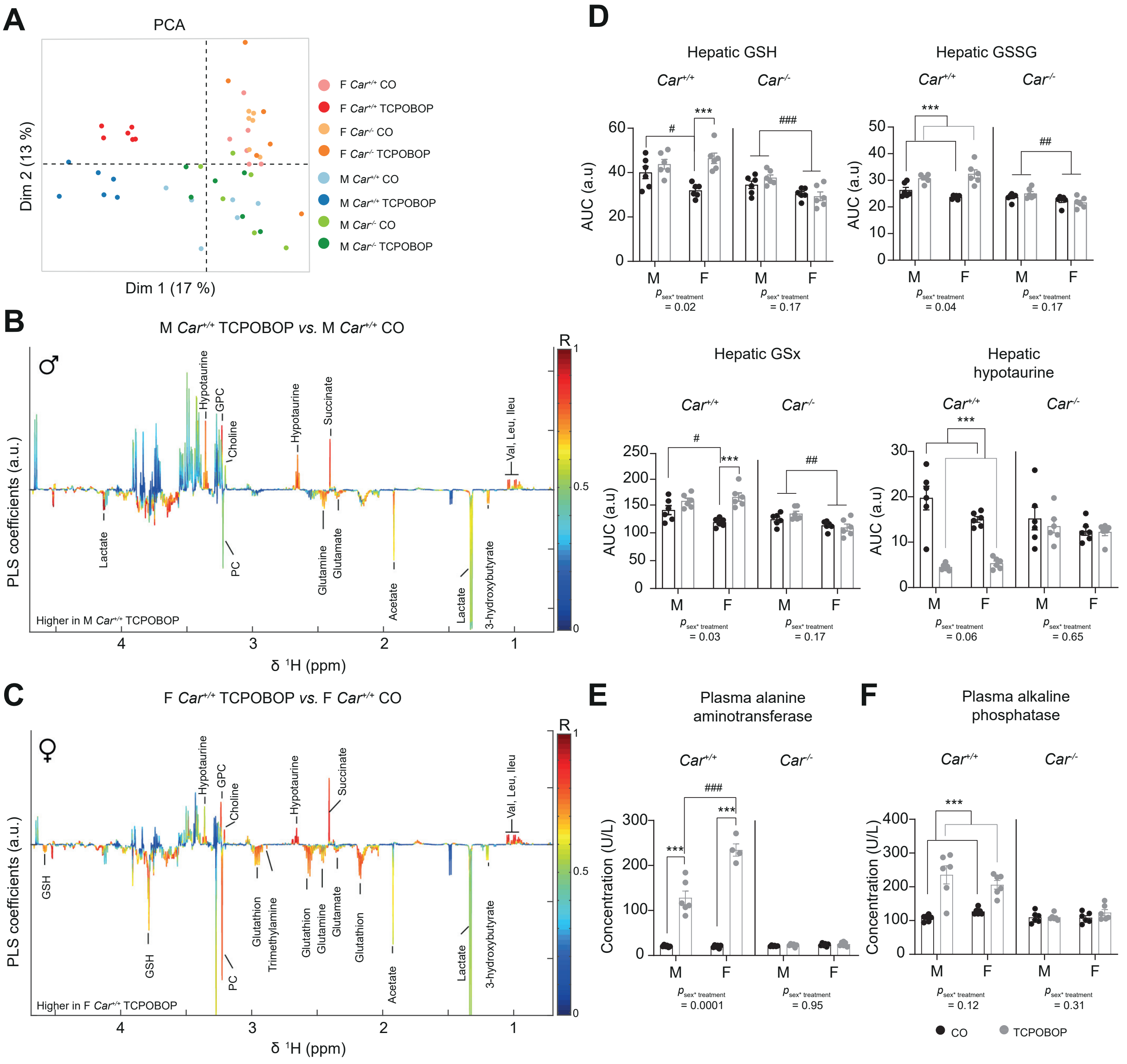

Fig. 4. TCPOBOP treatment increases liver oxidative stress and toxicity in a sex-biased manner. (A) PCA of the liver metabolomic dataset. (B) Coefficient plots related to the PLS-DA models discriminating liver extract spectra from TCPOBOP- and CO-treated *Car* $^{+/+}$ males. Parameters of the PLS-DA model: $Q^2Y = 0.89$, $p = 0.001$. (C) Coefficient plots related to the O-PLS-DA models discriminating between liver extract spectra from TCPOBOP- and CO-treated *Car* $^{+/+}$ females. Parameters of the PLS-DA model: $Q^2Y = 0.91$, $p = 0.001$. *(D)* AUC of the ^{1}H-NMR spectra was integrated for the glutathione signals (GSH, reduced form; GSSG, oxidised form; GSx, total glutathione) and for hypotaurine. (E) Plasma alanine aminotransferase. (F) Plasma alkaline phosphatase. Results are given as the mean ± SEM. *Treatment effect, #sex effect. * *or* #*p* <0.05, ** or ##*p* <0.01, *** *or* ###*p* <0.001 (two-way ANOVA). CAR, constitutive androstane receptor; CO, corn oil; GSH, reduced glutathione; GSSG, oxidized glutathione; GSx; PLS-DA, projection on latent structure-discriminant analysis; PCA, principal component analysis; TCPOBOP, 1,4-bis[2-(3,5-dichloropyridyloxy)] benzene.

Results are given as the mean ± SEM. *Treatment effect, #sex effect. * or #*p* <0.05, ** or ##*p* <0.01, *** or ###*p* <0.001 (two-way ANOVA). (E) Multi-omic integrative analysis performed on plasma metabolomic and hepatic transcriptomic datasets (DIABLO model). (F) Correlation network between hepatic transcripts and plasma metabolites (R^2 > 0.97, DIABLO model). (G) Fold-change (TCPOBOP- *vs.* CO-treated *Car* $^{+/+}$ mice) of hepatic expression for genes involved in lipoprotein metabolism. *Treatment effect, #sex effect. *p_{adj} <0.05, **p_{adj} <0.01, ***p_{adj} <0.001 (linear model). CAR, constitutive androstane receptor; CO, corn oil; PB, phenobarbital; PCA, principal component analysis; PLS-DA, orthogonal projection on latent structure-discriminant analysis; TCPOBOP, 1,4-bis[2-(3,5-dichloropyridyloxy)] benzene; Vldr, very low-density protein receptor.

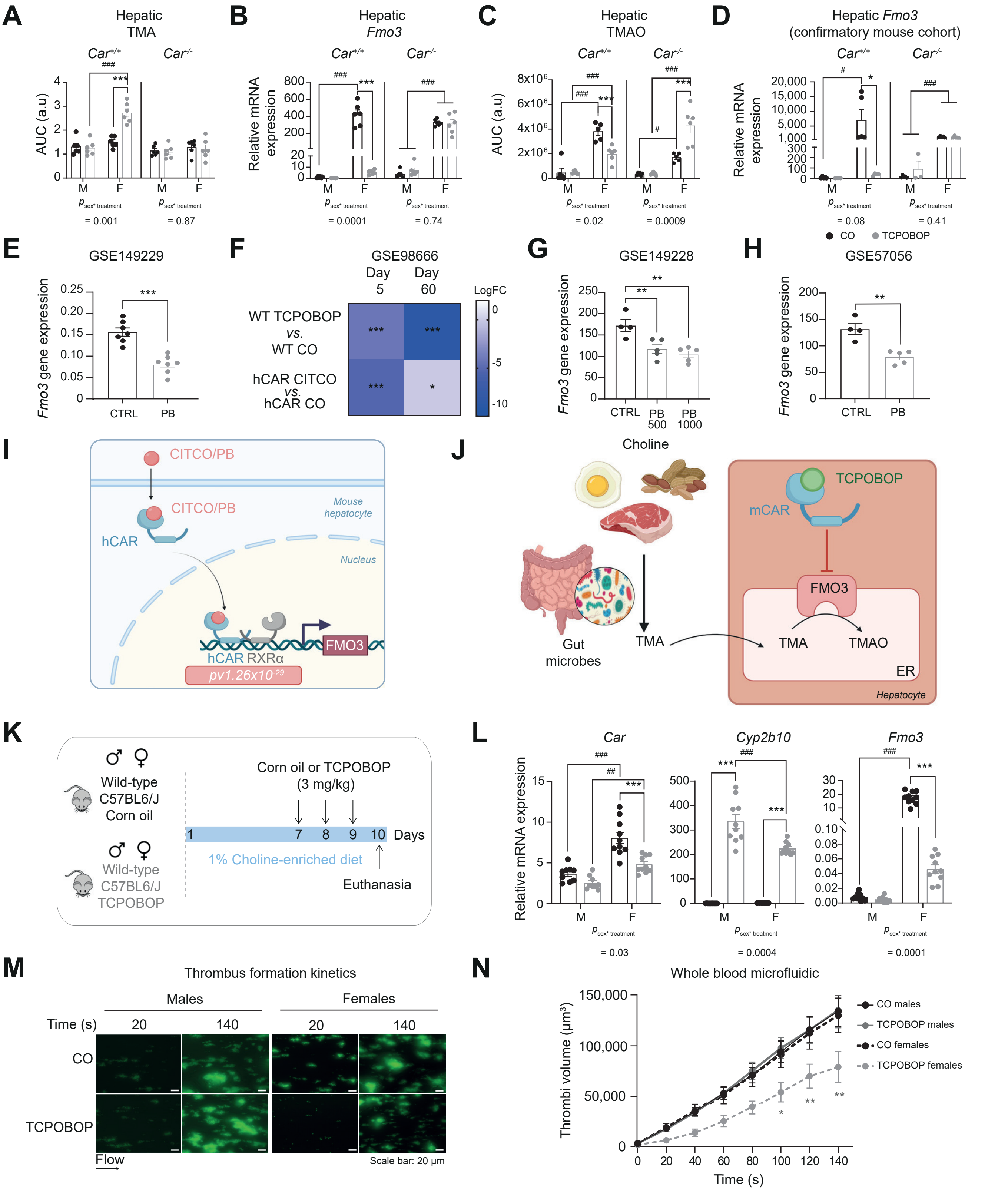

Fig. 5. CAR modulates liver TMA metabolism by regulating *Fmo3* gene expression, mostly in females. (A) AUC of the ^{1}H-NMR spectra for TMA. (B) Hepatic gene expression. (C) Hepatic content of TMAO. (D) Hepatic gene expression in independent confirmatory experiment. Results are mean ± SEM. *Treatment effect, #sex effect. * or #*p* <0.05, ** or ##*p* <0.01, *** or ### *p* <0.001 (two-way ANOVA). (E–H) Hepatic mRNA expression derived from publicly available datasets. **p* <0.05, ***p* <0.01, ****p* <0.001 (one-way ANOVA or Student *t* test). (I) Proposed model for direct binding of hCAR to Fmo3 regulatory DNA sequences based on Niu *et al.*[28] (created with BioRender.com). (J) Proposed impact of CAR activation on TMA metabolism (created with BioRender.com). (K) Experimental design. (L) Hepatic gene expression. Results are mean ± SEM. *Treatment effect, #sex effect. * or #*p* <0.05, ** or ##*p* <0.01, *** or ###*p* <0.001 (two-way ANOVA). (M) Representative images

Role of CAR in sexually-dimorphic regulation of hepatic gene expression in response to TCPOBOP

TCPOBOP impacted a much higher number of genes in the liver of females *vs.* males (~40% more DEGs in females *vs.* males, Fig. 1E). Accordingly, PCA of the microarray data projected on the second and third principal components showed a distinct clustering of TCPOBOP- and CO-treated females, whereas males from both groups were merged (Fig. 2A). To identify genes with sex-dependent regulation upon TCPOBOP, we next focused on the DEGs with a significant interaction between sex and treatment (Table S6). These 486 sex-specific DEGs clustered within four distinct expression profiles (Fig. 2B and C). Female-specific upregulated genes (cluster 1, 215 genes) were involved in xenobiotic metabolism (p = 10^{-12}) and extracellular matrix remodelling (p = 10^{-6}) and contained genes encoding for collagens (*Col4a1*), extracellular matrix degrading metalloproteinases (*Mmp12*, *Mmp13*) and proteinases involved in the processing of procollagens (*Adamst2*, *Adamst4*, *Adamst14*, and *Adamst15*), whereas female-specific downregulated genes (cluster 3, 170 genes) were involved in phase I xenobiotic metabolism (p = 10^{-8}) and flavin monooxygenase (FMO)-dependent oxidations (p = 10^{-6}) (Fig. 2D–F). As indicated above, male-specific CAR target genes were fewer. The male-specific upregulated genes (cluster 4, 37 genes) were involved in 'steroid metabolism' (p = $10^{-5.5}$), whereas no significantly enriched metabolic pathway was found using the male-specific downregulated genes (cluster 2, 64 genes) (Fig. 2D and E and Table S6). Overall, this analysis provided evidence that 10% of the TCPOBOP-sensitive genes were regulated in a sex-dependent manner (Fig. 2G).

CAR regulated plasma lipoprotein metabolism in a sex-independent manner

We next explored the systemic consequences of CAR activation using plasma metabolomics. PCA analysis of the whole plasma metabolic profiles showed a separation of male *vs.* female mice on the first principal component, illustrating a constitutive sexual dimorphism in plasma metabolite levels, while TCPOBOP-treated *Car*$^{+/+}$ were discriminated from *Car*$^{-/-}$ mice and from *Car*$^{+/+}$ CO-treated mice on the second principal component, illustrating a significant effect of TCPOBOP on plasma metabolites (Fig. 3A). As seen for hepatic transcripts, there was no significant differences between metabolic profiles from TCPOBOP *vs.* CO-treated *Car*$^{-/-}$ mice (Fig. S3). In *Car*$^{+/+}$ males, TCPOBOP treatment decreased glucose, increased lactate levels, and had a major impact on circulating lipoproteins: cholesterol and several broad lipid peaks were strongly decreased upon TCPOBOP treatment, while other lipid peaks were increased (Fig. 3B). In *Car*$^{+/+}$ females, cholesterol and lipid signals were changed in a similar manner than in males (Fig. 3C). Putative assignment of these differential peaks revealed that the decreased cholesterol peak reflected HDL-cholesterol, decreased lipid peaks belonged to LDLs and increased lipids belonged to VLDLs. The area under the curve for selected HDL-cholesterol, lipid-LDL and lipid-VLDL signals further illustrated this CAR-dependent impact of TCPOBOP treatment on circulating lipoproteins in both sexes (Fig. 3D). Total plasma-, HDL-cholesterol, plasma triglycerides, free fatty acids, and glucose were quantified through additional classical biochemical assays and confirmed the strong CAR-dependent impact of TCPOBOP on plasma metabolites observed by metabolomics (Fig. S4).

Plasma metabolomics and hepatic transcriptomic data statistical integration revealed a strong correlation between plasma metabolites and liver transcripts (R^2 = 0.94) regardless of sex (Fig. 3E). The correlation network highlighted a strong positive correlation between *Apoc1* mRNA and plasma LDL-cholesterol and HDL-cholesterol (Fig. 3F). This led us to investigate more closely the hepatic expression of genes involved in hepatic cholesterol metabolism (Fig. 3G). CAR activation significantly decreased the expression of most apolipoproteins. Expression of the *Ldl receptor* (*Ldlr*), which is responsible for LDL clearance was unchanged; however, the expression of *Vld receptor* (*Vldlr*) was increased by a factor of four upon TCPOBOP treatment. *Mttp*, the protein that transports triglycerides and cholesterol esters in the endoplasmic reticulum for VLDL synthesis was also significantly increased. Moreover, TCPOBOP treatment impacted cholesterol and bile acid metabolism with decreased expression of genes involved in cholesterol transport, decreased expression of genes involved in bile acid synthesis and increased expression of genes involved in bile acid detoxification and transport. All significant changes in hepatic mRNA and plasma metabolites related to cholesterol metabolism were CAR-dependent and were similar in both sexes (Fig. S5 and S6). Altogether, these results illustrate that CAR activation deeply modulates hepatic and systemic cholesterol metabolism in a sex-independent way.

TCPOBOP induced liver oxidative stress and toxicity in a sex-biased way

We next performed metabolic profiling of hydrophilic metabolites in liver tissue. PCA of the entire metabolic profile revealed a distinct clustering of *Car*$^{+/+}$ mice treated with TCPOBOP *vs.* all other mouse groups on the first principal component, whereas male and female mice were separated on the second component, revealing once again a major impact of CAR activation on liver metabolites (Fig. 4A). TCPOBOP-treated males displayed significant changes in hepatic levels of many amino-acids (increased glutamine and glutamate and decreased valine, leucine, and isoleucine), energy-related metabolites (increased lactate and 3-hydroxybutyrate, and decreased succinate), cell membrane constituents (decreased choline and glycerophosphocholine and increased phosphocholine) and metabolites involved in oxidative stress (decreased hypotaurine) (Fig. 4B). In females, most of these metabolites followed the same pattern, with perturbations of metabolites involved in oxidative stress being more pronounced than in males (significant increased levels of reduced [GSH], oxidised [GSSG], and total [Gsx] glutathione) (Fig. 4C). AUC for glutathione signals illustrated that TCPOBOP induced a more pronounced hepatic oxidative stress in females than in males (Fig. 4D). All significant changes in hepatic metabolites related to oxidative stress upon TCPOBOP were CAR-dependent (Fig. S7). Finally, sex-dependent TCPOBOP hepatic toxicity was confirmed through biochemical quantification of plasmatic markers (Fig. 4E–F). Circulating levels of ALT were significantly higher in TCPOBOP-treated *Car*$^{+/+}$ females *vs.* males, whereas plasma ALP was significantly increased in both sexes upon

of platelet adhesion. (N) Quantification of platelet adhesion to a microfluidic chip surface *p <0.05, **p <0.01 (one-way ANOVA). CAR, constitutive androstane receptor; GSH, reduced glutathione; GSSG, oxidised glutathione; GSx, total glutathione; PCA, principal component analysis; PLS-DA, orthogonal projection on latent structure-discriminant analysis; TMA, trimethylamine; TMAO, trimethylamine N-oxide.

TCPOBOP with a tendency to higher levels in males ($P_{sex*treatment} = 0.12$). Overall, our results demonstrate a sex-dependent impact of CAR activation on liver metabolism and on the toxicity profile.

CAR modulated liver trimethylamine metabolism through regulation of *Fmo3* gene expression mostly in females

Another intriguing sex-dependent hepatic impact of TCPOBOP was the female-specific, CAR-dependent increased level of trimethylamine (TMA) (Fig. 4C and Fig. 5A). TMA is a gut microbiota-dependent metabolite that is metabolised to TMAO by the liver-specific flavin monooxygenase 3 (FMO3).[23] This result was in accordance with the female-specific decrease of *Fmo3* mRNA expression observed previously in the microarray data (Fig. 2E) and was confirmed here with RT-qPCR (Fig. 5B). Finally, we quantified hepatic TMAO and confirmed a twofold decrease of this metabolite in TCPOBOP-treated *Car*$^{+/+}$ females compared with vehicle-treated females (Fig. 5C). Female-biased inhibition of *Fmo3* mRNA by CAR activation was reproducible in an independent study in which mice were treated with TCPOBOP or CO every 2 days for 10 days (Fig. 5D). Next, we analysed *Fmo3* hepatic expression in several publicly available gene expression datasets. In the first experiment, *Car*$^{-/-}$ mice were knocked-in with human CAR coding sequence[24] and were fed diets containing 0 (CTRL) or 1,000 ppm PB, an indirect activator of both mCAR and hCAR.[25] We found that PB-fed hCAR mice had significantly lower expression of hepatic *Fmo3* mRNA compared with control mice (Fig. 5E). The second study compared wild-type (WT) mice treated with TCPOBOP *vs.* CO-treated mice and hCAR mice treated with CITCO (a specific agonist of hCAR) *vs.* CO-treated hCAR mice.[26] Both TCPOBOP and CITCO-treated mice had significantly lower *Fmo3* hepatic mRNA compared with their relative controls (Fig. 5F). Finally, the last two studies were conducted in chimeric mice with human hepatocytes treated with PB.[25,27] Again, we found a significant decrease in *Fmo3* hepatic gene expression in response to PB in both datasets (Fig. 5G and 5H). It is worth noting that all publicly available studies were conducted in male mice only, which could explain why the decrease in *Fmo3* expression seen upon CAR activation by PB or CITCO was of lower magnitude than that seen in our own *in vivo* experiments in females. Finally, we took advantage of the only available ChIP-seq analysis of hCAR binding *in vivo* to date[28] and observed that, among the 6,364 unique genes associated with high-confidence hCAR-binding genes, *Fmo3* was found as a hCAR-direct binding gene with a *p value* = 1.26×10^{-29} (Fig. 5I). Thus, in females, CAR activation by TCPOBOP perturbated the metabolism of TMAO from TMA by inhibiting the expression of *Fmo3* (Fig. 5J) and this result might be relevant to humans.

CAR activation decreased platelet hyperactivity induced by dietary choline supplementation

FMO3 activity and TMAO levels have been shown to modulate platelet hyper-responsiveness and thrombosis potential.[29,30] We thus wondered whether CAR activation could also influence platelet function. To enhance platelet responsiveness, female and male mice were fed a choline-enriched diet before treatment with TCPOBOP (Fig. 5K). We confirmed significant hepatic CAR activation in both sexes (Fig. 5L) and examined thrombi formation. Whole blood from female mice treated with TCPOBOP formed smaller thrombi over time compared with blood from control mice. In males, TCPOBOP treatment did not affect thrombi formation. Thus, *in vivo* TCPOBOP treatment modulates platelet activation and reduces the thrombotic risk of females specifically (Fig. 5M and N).

Discussion

The liver appears to be one of the most sexually dimorphic organs and expression of genes involved in drug metabolism is sexually dimorphic in rodents and humans.[8,31] CAR is the target of many drugs and is widely involved in the control of expression of xenobiotic metabolising genes. However, a genome-wide description of sex-specific CAR-dependent sensitive genes was lacking. Here, we provide an exhaustive study of the transcriptomic impact of acute pharmacological CAR activation in male *vs.* female mice and novel insights into the metabolic impact of this activation.

First, most TCPOBOP-modulated genes were regulated in a similar manner in male and female livers, especially those involved in the cell cycle. Our results are consistent with other studies revealing that chronic activation of CAR using TCPOBOP promotes tumour formation in rodents in a CAR-dependent manner.[32–34] The underlying mechanisms depend on direct CAR-dependent induction of *Mdm2*, a primary inhibitor of P53 and induction of the transcription factor *FoxM1*,[35] which is essential for the initiation of carcinogen-induced liver tumours, thus resulting in modulation of many genes implicated in cell proliferation, cellular growth, apoptosis, and cell differentiation, such as *Gadd45b*, *Ccnd1*, *Ccnb1*, *C-myc*, and *Yap*.[35,36] Few studies have highlighted that sex could influence TCPOBOP-induced liver proliferation but showed inconsistent results. Some studies described that female mice were more sensitive to TCPOBOP-induced liver proliferation,[12,37] whereas others showed no tumour development in female mice treated with the genotoxin diethylnitrosamine followed by TCPOBOP, compared with males.[38] Similarly, after a single injection of TCPOBOP, male mice displayed a deeper disturbance of key cell cycle genes.[16] Unlike these studies, we did not reveal any sexual dimorphism on hepatomegaly and cell cycle gene modulation upon CAR activation. However, a long-term analysis of TCPOBOP-induced liver tumours conducted in male and female mice in parallel would be required to further investigate this discrepancy.

We next observed a strong impact of TCPOBOP treatment on plasma lipoproteins, with decreased total-, HDL- and LDL-cholesterol measured in both sexes. This result is consistent with previous findings whereby TCPOBOP decreased circulating levels of plasma HDL in both WT and transgenic mice expressing human apolipoprotein A-1, at least in part through down-regulation of *ApoA-1* hepatic gene expression.[39] Similarly, in *Ldlr*$^{-/-}$ mice fed a Western-diet, TCPOBOP decreased circulating levels of ApoB-containing lipoproteins (mainly VLDL and LDL) and reduced the development of atherosclerotic lesions,[40] presumably through CAR-mediated induction of the VLDL receptor, a receptor involved in the clearance of VLDL and LDL as a backup for the LDL receptor.[41] Here, we confirm that CAR activation results in decreased *ApoA-1* and increased *Vldlr* hepatic mRNA levels, which could both participate to the observed decrease of plasmatic HDL and LDL levels. Moreover, we also observed a strong decrease in hepatic expression of other major lipoprotein-coding genes (namely *ApoB*, *Apoc1*, and *ApoE*), and of *Lecithin cholesterol acyl transferase* (*Lcat*, another constitutive component of HDL) which could also play a role.

Another well-known function of CAR is its ability to promote bile acid detoxification during cholestasis.[42,43] As previously

described, we found that the expression of genes involved in hydroxylation, sulfation, and excretion of bile acids was significantly enhanced upon CAR activation, whereas expression of genes involved in bile acid synthesis and cholesterol transport was decreased in both sexes. The emerging role of CAR in cholesterol homeostasis represents new perspectives in the treatment of hypercholesterolaemia and atherosclerosis.[39,44] The current study confirms and extends these previous studies reporting the effects of TCPOBOP on hepatic expression of genes involved in bile acid, cholesterol and lipoprotein metabolism, as well as those on lipoprotein concentrations, are sex-independent, at least in mice. Our findings may have clinical relevance. Indeed, a recent study combining genome-wide analysis of cholestatic mice genetic models and data-mining of human patient cohorts with various liver diseases unravelled a significant enrichment of CAR-sensitive genes in cholestatic livers specifically.[45] Moreover, CAR activation in cholestasis leads to alterations of drug metabolism with significant effects on drug-induced hepatotoxicity. Drug-induced liver injury (DILI) is still a serious clinical concern and one of the most common drug adverse reactions. DILI clinical phenotype is influenced by age and sex.[46,47] Here, we found that, upon TCPOBOP, 385 genes displayed a female-specific *vs.* 101 genes with a male-specific response. Many female-specific genes were involved in extracellular matrix organisation. We also observed stronger perturbations of hepatic metabolites involved in glutathione metabolism in livers of females compared with males, reflecting higher hepatic oxidative stress. Finally, we highlighted a sexually dimorphic impact of CAR activation on clinical markers of liver toxicity, namely significantly higher levels of ALT in females compared with males, and a trend toward higher ALP levels in males. This result is consistent with the sex-influence on DILI clinical phenotype with cytolytic damage being more frequently observed in women, whereas cholestatic damage presented a male predominance.[46,47] It is well known that women experience higher rates,[48] and more severe[49] adverse drug reactions than men. However, mechanistic explanations for these observations are often lacking. Our present results suggest that drugs interacting with CAR may be considered with particular attention before their use in women.

Limitations of our study include the use of only one drug (TCPOBOP), whereas DILI has been shown to depend both on patient characteristics and on drug properties.[50] Our results therefore need to be confirmed with other drugs that act as CAR agonists.

Another novel finding from our study was the strong increase in hepatic TMA upon TCPOBOP administration observed in female mice specifically. TMA is a product of the gut microbial metabolism of phosphatidylcholine, choline, and L-carnitine. It is transported from the gut to the liver via the portal vein and N-oxidised into TMAO by host FMO3.[51] Analysis of natural genetic variation in inbred strains of mice indicate that FMO3 and TMAO are significantly correlated and explain more than 10% of the variation in atherosclerosis.[51] Since then, it has been confirmed that high circulating levels of TMAO are linked to increased thrombotic and cardiovascular risks in animal and human studies, even after adjustment for known cardiovascular risk factors.[52,53] Consistent with increased TMA, hepatic *Fmo3* mRNA expression and TMAO concentration were both strongly decreased in *Car*$^{+/+}$ females treated with TCPOBOP. We confirmed the CAR-dependent regulation of *Fmo3* mRNA in publicly available cohorts that used different hCAR models and different mCAR and hCAR agonists, therefore suggesting that the regulation of *Fmo3* expression is not dependent on the CAR agonist used and might be relevant in humans. In rodents, hepatic *Fmo3* knockdown was sufficient to decrease diet-dependent platelet responsiveness and thrombotic potential.[29,30] Here, we observed that, in conditions of diet-induced platelet hyper-responsiveness, CAR activation was indeed sufficient to significantly modulate thrombus growth in female mice specifically. We postulate that this effect is, at least in part, attributable to the CAR-mediated downregulation of *Fmo3* expression and activity in females. Nowadays, drugs represent the main cause of platelet dysfunction.[54,55] Our results suggest that drugs or other xenobiotics (such as pollutants, foods) that interact with CAR could decrease thrombus formation in a pro-thrombotic context. These compounds may provide a beneficial effect by modulating platelet activation and thrombosis. We, therefore, highlight a new axis between hepatic xenobiotic metabolism and blood haemostasis. We suggest that this axis may be especially relevant in women. However, there are important species-specific sex-based differences in *FMO3* expression: its expression is female-specific in mice as a result of modulation by sex steroids,[56] whereas its abundance was significantly associated with females in humans but to a much lower extent than in rodents.[57] Thus, the gender-specificity and clinical relevance of this CAR–FMO3–TMAO–platelet axis deserves further investigation. In addition to platelet function and thrombotic risk, an increase in the TMAO plasma concentration has also been shown to increase the risk of impaired glucose tolerance,[58] colorectal cancer,[59] chronic kidney disease,[60] and overall mortality.[61] Whether drugs interacting with CAR could also influence these TMAO-dependent endpoints deserves further investigations.

In summary, the present study provides an exhaustive description of the sex-independent and sex-dependent CAR-sensitive genes and demonstrates a stronger impact of CAR pharmacological activation on hepatic transcriptome and metabolism of the female. Additionally, CAR activation impacted the TMA–FMO3–TMAO pathway in females, which might link drugs and environmental xenobiotic exposure with platelet aggregation and other TMAO-sensitive physiological responses.

Abbreviations

^{1}H-NMR, proton nuclear magnetic resonance; ALP, alkaline phosphatase; ALT, alanine aminotransferase; CAR, constitutive androstane receptor; ChIP-seq, chromatin immunoprecipitation sequencing; CITCO, 6-(4-Chlorophenyl)imidazo[2,1-b][1,3]thiazole-5-carbaldehyde-O-(3,4-dichlor obenzyl)oxime; CO, corn oil; CYPs, cytochromes P450; Cy3, cyanine3; DEGs, differentially-expressed genes; DILI, drug-induced liver injury; DIOC6, 3,3′-dihexyloxacarbocyanine iodide; FC, fold change; FDR, false discovery rate; FMO3, flavin monooxygenase 3; GEO, gene expression omnibus; GO, gene ontology; GSH, reduced glutathione; GSSG, oxidised glutathione; GSx, total glutathione; hCAR, humanised constitutive androstane receptor; HSQC, heteronuclear single quantum coherence; Lcat, lecithin cholesterol acyl transferase; Ldlr, low-density lipoprotein receptor; mCAR, murine constitutive androstane receptor; MRM, multiple reaction monitoring; Mttp, microsomal triglyceride transfer protein; O-PLS-DA, orthogonal projection on latent structure-discriminant analysis;

PB, phenobarbital; PCA, principal component analysis; RT-qPCR, real-time quantitative polymerase chain reaction; Tbp, TATA-box-binding protein; TCPOBOP, 1,4-bis[2-(3,5-dichloropyridyloxy)] benzene; TMA, trimethylamine; TMAO, trimethylamine N-oxide; Vldr, very low-density protein receptor; WAT, perigonadal white adipose tissue; WT, wild-type; ZT, zeitgeber time.

Financial support

MH is the recipient of a PhD grant from the French Ministry of Research. This work was supported by Région Occitanie (Hepatomics to NL) the Fondation pour la Recherche Médicale (grant number ENV202109013962 to HG) and grants from the French National Research Agency (Hepatomorphic ANR-20-CE14-0035 to HG; Gadget ANR-22-CE34-0005-01 to SE-S).

Conflicts of interest

The authors declare no conflicts of interest.

Please refer to the accompanying ICMJE disclosure forms for further details.

Authors' contributions

Conceptualisation: MH, FL, NL, SE-S.Methodology: MH, FL, MG, BE, AP, CC. Software and statistical analysis: MH, AP, YL, SE-S. Investigation: MH, FL, MG, BE, JB, TF, CM, CR, AF, CN, GG, ER-B, SE-S. Writing—original draft preparation: MH, SE-S. Writing—review and editing: MH, BE, AF, ER-B, UR-K, BP, EB-R, LG-P, HG, NL, SE-S. Supervision: YL, ER-B, LD, UR-K, MB, BP, NL, HG, SE-S. Project administration, NL, HG, SE-S. Funding acquisition: MB, BP, NL, LG-P, HG, SE-S. Read and approved the final manuscript: all authors.

Data availability statement

Gene expression data generated for this study with microarray are available in NCBI's Gene Expression Omnibus and are accessible through GEO Series accession number GSE228554. Other data are available from the corresponding author upon reasonable request.

Acknowledgements

We thank all members of the EZOP (Experimental Zootechny), UMR Toxalim, Toulouse, France, staff for their careful help with this project. We also thank the staff from the Génotoul: Anexplo, Get-TriX, and Metatoul-AXIOM facilities.

Supplementary data

Supplementary data to this article can be found online at https://doi.org/10.1016/j.jhepr.2023.100930.

References

Author names in bold designate shared co-first authorship

[1] Wei P, Zhang J, Egan-Ha M, Moore DD. The nuclear receptor CAR mediates specific xenobiotic induction of drug metabolism. Nature 2000;407:920–923.
[2] Mackowiak B, Hodge J, Stern S, Wang H. The roles of xenobiotic receptors: beyond chemical disposition. Drug Metab Dispos 2018;46:1361–1371.
[3] Moore LB, Parks DJ, Jones SA, et al. Orphan nuclear receptors constitutive androstane receptor and pregnane X receptor share xenobiotic and steroid ligands. J Biol Chem 2000;275:15122–15127.
[4] **Huang W**, **Zhang J**, Chua SS, et al. Induction of bilirubin clearance by the constitutive androstane receptor (CAR). Proc Natl Acad Sci U S A 2003;100:4156–4161.
[5] Honkakoski P, Zelko I, Sueyoshi T, Negishi M. The nuclear orphan receptor CAR-retinoid X receptor heterodimer activates the phenobarbital-responsive enhancer module of the *CYP2B* gene. Mol Cell Biol 1998;18:5652–5658.
[6] Maglich JM, Stoltz CM, Goodwin B, et al. Nuclear pregnane X receptor and constitutive androstane receptor regulate overlapping but distinct sets of genes involved in xenobiotic detoxification. Mol Pharmacol 2002;62:638–646.
[7] **Hernandez J**, Mota L, Baldwin W. Activation of CAR and PXR by dietary, environmental and occupational chemicals alters drug metabolism, intermediary metabolism, and cell proliferation. Curr Pharmacogenomics Pers Med 2009;7:81–105.
[8] Lamba V, Lamba J, Yasuda K, et al. Hepatic CYP2B6 expression: gender and ethnic differences and relationship to *CYP2B6* genotype and CAR (Constitutive Androstane Receptor) expression. J Pharmacol Exp Ther 2003;307:906–922.
[9] Lu Y-F, Jin T, Xu Y, et al. Sex differences in the circadian variation of cytochrome P450 genes and corresponding nuclear receptors in mouse liver. Chronobiol Int 2013;30:1135–1143.
[10] Hernandez JP, Huang W, Chapman LM, et al. The environmental estrogen, nonylphenol, activates the constitutive androstane receptor. Toxicol Sci 2007;98:416–426.
[11] Tzameli I, Pissios P, Schuetz EG, Moore DD. The xenobiotic compound 1,4-Bis[2-(3,5-dichloropyridyloxy)]benzene is an agonist ligand for the nuclear receptor CAR. Mol Cell Biol 2000;20:2951–2958.
[12] Ledda-Columbano GM. Sex difference in the proliferative response of mouse hepatocytes to treatment with the CAR ligand, TCPOBOP. Carcinogenesis 2003;24:1059–1065.
[13] Lukowicz C, Ellero-Simatos S, Régnier M, et al. Dimorphic metabolic and endocrine disorders in mice lacking the constitutive androstane receptor. Sci Rep 2019;9:20169.
[14] **Hernandez J**, **Mota L**, Huang W, et al. Sexually dimorphic regulation and induction of P450s by the constitutive androstane receptor (CAR). Toxicology 2009;256:53–64.
[15] Geter DR, Bhat VS, Gollapudi BB, et al. Dose-response modeling of early molecular and cellular key events in the car-mediated hepatocarcinogenesis pathway. Toxicol Sci 2014;138:425–445.
[16] Lodato NJ, Melia T, Rampersaud A, Waxman DJ. Sex-differential responses of tumor promotion-associated genes and dysregulation of novel long noncoding RNAs in constitutive androstane receptor-activated mouse liver. Toxicol Sci 2017;159:25–41.
[17] Goldfarb CN, Waxman DJ. Global analysis of expression, maturation and subcellular localization of mouse liver transcriptome identifies novel sex-biased and TCPOBOP-responsive long non-coding RNAs. BMC Genomics 2021;22:212.
[18] Lukowicz C, Ellero-Simatos S, Régnier M, et al. Metabolic effects of a chronic dietary exposure to a low-dose pesticide cocktail in mice: sexual dimorphism and role of the constitutive androstane receptor. Environ Health Perspect 2018;126:067007.
[19] Huang DW, Sherman BT, Lempicki RA. Bioinformatics enrichment tools: paths toward the comprehensive functional analysis of large gene lists. Nucleic Acids Res 2009;37:1–13.
[20] Lê Cao K-A, González I, Déjean S. integrOmics: an R package to unravel relationships between two omics datasets. Bioinformatics 2009;25:2855–2856.
[21] Mahi NA, Najafabadi MF, Pilarczyk M, et al. GREIN: an interactive web platform for re-analyzing GEO RNA-seq data. Sci Rep 2019;9:7580.
[22] Laurent P-A, Séverin S, Hechler B, et al. Platelet PI3Kβ and GSK3 regulate thrombus stability at a high shear rate. Blood 2015;125:881–888.
[23] Lang D, Yeung C, Peter R, et al. Isoform specificity of trimethylamine N-oxygenation by human flavin-containing monooxygenase (FMO) and P450 enzymes. Biochem Pharmacol 1998;56:1005–1012.
[24] Zhang Y-KJ, Lu H, Klaassen CD. Expression of human CAR splicing variants in BAC-transgenic mice. Toxicol Sci 2013;132:142–150.
[25] Yamada T, Okuda Y, Kushida M, et al. Human hepatocytes support the hypertrophic but not the hyperplastic response to the murine non-genotoxic hepatocarcinogen sodium phenobarbital in an in vivo study using a chimeric mouse with humanized liver. Toxicol Sci 2014;142:137–157.
[26] Cheng SL, Bammler TK, Cui JY. RNA-seq reveals age- and species differences of CAR-targeted drug processing genes. Drug Metab Dispos 2017:867–882.
[27] Yamada T, Ohara A, Ozawa N, et al. Comparison of the hepatic effects of phenobarbital in chimeric mice containing either rat or human hepatocytes with humanized constitutive androstane receptor (CAR) and pregnane X receptor (PXR) mice (hCAR/hPXR mice). Toxicol Sci 2020:362–376.
[28] Niu B, Coslo DM, Bataille AR, et al. In vivo genome-wide binding interactions of mouse and human constitutive androstane receptors reveal novel gene targets. Nucleic Acids Res 2018;46:8385–8403.
[29] **Zhu W**, **Gregory JC**, Org E, et al. Gut microbial metabolite TMAO enhances platelet hyperreactivity and thrombosis risk. Cell 2016;165:111–124.
[30] Zhu W, Buffa JA, Wang Z, et al. Flavin monooxygenase 3, the host hepatic enzyme in the metaorganismal trimethylamine N-oxide-generating pathway, modulates platelet responsiveness and thrombosis risk. J Thromb Haemost 2018;16:1857–1872.

[31] Conforto TL, Waxman DJ. Sex-specific mouse liver gene expression: genome-wide analysis of developmental changes from pre-pubertal period to young adulthood. Biol Sex Differ 2012;3:9.
[32] Dragani TA, Giacomo M, Galliani G, Della Porta G. Promoting effects of l,4-bis[2-(3,5-dichloropyridyloxy)]benzene in mouse hepatocarcinogenesis. Carcinogenesis 1985:225–228.
[33] Diwan BA, Lubet RA, Ward JM, et al. Tumor-promoting an d hepatocarcinogenic effects of l,4-bis[2-(3,5-dichloropyridyloxy)]benzene (TCPOBOP) in DBA/2NCr and C57BL/6NO mice and an apparent promoting effect on nasal cavity tumors but not on hepatocellular tumors in F344/NO rats initiated with 7V-nitrosodiethylamine. Carcinogenesis 1992:1893–1901.
[34] **Huang W**, **Zhang J**, Washington M, et al. Xenobiotic stress induces hepatomegaly and liver tumors via the nuclear receptor constitutive androstane receptor. Mol Endocrinol 2005;19:1646–1653.
[35] **Blanco-Bose WE**, **Murphy MJ**, Ehninger A, et al. c-Myc and its target FoxM1 are critical downstream effectors of constitutive androstane receptor (CAR) mediated direct liver hyperplasia. Hepatology 2008:1302–1311.
[36] Kazantseva YA, Yarushkin AA, Pustylnyak VO. CAR-mediated repression of Foxo1 transcriptional activity regulates the cell cycle inhibitor p21 in mouse livers. Toxicology 2014;321:73–79.
[37] Ganzenberg K, Singh Y, Braeuning A. The time point of β-catenin knockout in hepatocytes determines their response to xenobiotic activation of the constitutive androstane receptor. Toxicology 2013;308:113–121.
[38] Li Z, Tuteja G, Schug J, Kaestner KH. Foxa1 and Foxa2 Are essential for sexual dimorphism in liver cancer. Cell 2012;148:72–83.
[39] Masson D, Qatanani M, Sberna AL, et al. Activation of the constitutive androstane receptor decreases HDL in wild-type and human apoA-I transgenic mice. J Lipid Res 2008;49:1682–1691.
[40] Sberna A-L, Assem M, Xiao R, et al. Constitutive androstane receptor activation decreases plasma apolipoprotein B–containing lipoproteins and atherosclerosis in low-density lipoprotein receptor–deficient mice. Arterioscler Thromb Vasc Biol 2011;31:2232–2239.
[41] Degrace P, Moindrot B, Mohamed I, et al. Upregulation of liver VLDL receptor and FAT/CD36 expression in LDLR−/− apoB100/100 mice fed trans-10,cis-12 conjugated linoleic acid. J Lipid Res 2006;47:2647–2655.
[42] Guo GL, Lambert G, Negishi M, et al. Complementary roles of farnesoid X receptor, pregnane X receptor, and constitutive androstane receptor in protection against bile acid toxicity. J Biol Chem 2003;278:45062–45071.
[43] Stedman CAM, Liddle C, Coulter SA, et al. Nuclear receptors constitutive androstane receptor and pregnane X receptor ameliorate cholestatic liver injury. Proc Natl Acad Sci U S A 2005;102:2063–2068.
[44] Stern S, Kurian R, Wang H. Clinical relevance of the constitutive androstane receptor. Drug Metab Dispos 2022;50:1010–1018.
[45] Mathur B, Arif W, Patton ME, et al. Transcriptomic analysis across liver diseases reveals disease-modulating activation of constitutive androstane receptor in cholestasis. JHEP Rep 2020;2:100140.
[46] Lucena MI, Andrade RJ, Kaplowitz N, et al. Phenotypic characterization of idiosyncratic drug-induced liver injury: the influence of age and sex. Hepatology 2009;49:2001–2009.
[47] Suzuki A, Barnhart H, Gu J, et al. Associations of gender and a proxy of female menopausal status with histological features of drug-induced liver injury. Liver Int 2017;37:1723–1730.
[48] Soldin OP, Chung SH, Mattison DR. Sex differences in drug disposition. J Biomed Biotechnol 2011;2011:1–14.
[49] Kando JC, Yonkers KA, Cole JO. Gender as a risk factor for adverse events to medications. drugs 1995;50:1–6.
[50] George N, Chen M, Yuen N, et al. Interplay of gender, age and drug properties on reporting frequency of drug-induced liver injury. Regul Toxicol Pharmacol 2018;94:101–107.
[51] Bennett BJ, de Aguiar Vallim TQ, Wang Z, et al. Trimethylamine-N-oxide, a metabolite associated with atherosclerosis, exhibits complex genetic and dietary regulation. Cell Metab 2013;17:49–60.
[52] Wang Z, Klipfell E, Bennett BJ, et al. Gut flora metabolism of phosphatidylcholine promotes cardiovascular disease. Nature 2011;472:57–63.
[53] Koeth RA, Wang Z, Levison BS, et al. Intestinal microbiota metabolism of l-carnitine, a nutrient in red meat, promotes atherosclerosis. Nat Med 2013;19:576–585.
[54] Konkle BA. Acquired disorders of platelet function. Hematology Am Soc Hematol Educ Program 2011;2011:391–396.
[55] Scharf R. Drugs that affect platelet function. Semin Thromb Hemost 2012;38:865–883.
[56] Falls JG, Ryu D-Y, Cao Y, et al. Regulation of mouse liver flavin-containing monooxygenases 1 and 3 by sex steroids. Arch Biochem Biophys 1997;342:212–223.
[57] Xu M, Bhatt DK, Yeung CK, et al. Genetic and nongenetic factors associated with protein abundance of flavin-containing monooxygenase 3 in human liver. J Pharmacol Exp Ther 2017;363:265–274.
[58] Miao J, Fang S, Bae Y, Kemper JK. Functional inhibitory cross-talk between constitutive androstane receptor and hepatic nuclear factor-4 in hepatic lipid/glucose metabolism is mediated by competition for binding to the DR1 Motif and to the common coactivators, GRIP-1 and PGC-1α. J Biol Chem 2006;281:14537–14546.
[59] Xu R, Wang Q, Li L. A genome-wide systems analysis reveals strong link between colorectal cancer and trimethylamine N-oxide (TMAO), a gut microbial metabolite of dietary meat and fat. BMC Genomics 2015;16:S4.
[60] Hu DY, Wu MY, Chen GQ, et al. Metabolomics analysis of human plasma reveals decreased production of trimethylamine N-oxide retards the progression of chronic kidney disease. Br J Pharmacol 2022;179:4344–4359.
[61] Li D, Lu Y, Yuan S, et al. Gut microbiota–derived metabolite trimethylamine-N-oxide and multiple health outcomes: an umbrella review and updated meta-analysis. Am J Clin Nutr 2022;116:230–243.

Journal of Hepatology, Volume *6*

Supplemental information

Pharmacological activation of constitutive androstane receptor induces female-specific modulation of hepatic metabolism

Marine Huillet, Frédéric Lasserre, Marie-Pierre Gratacap, Beatrice Engelmann, Justine Bruse, Arnaud Polizzi, Tiffany Fougeray, Céline Marie Pauline Martin, Clémence Rives, Anne Fougerat, Claire Naylies, Yannick Lippi, Géraldine Garcia, Elodie Rousseau-Bacquie, Cécile Canlet, Laurent Debrauwer, Ulrike Rolle-Kampczyk, Martin von Bergen, Bernard Payrastre, Elisa Boutet-Robinet, Laurence Gamet-Payrastre, Hervé Guillou, Nicolas Loiseau, and Sandrine Ellero-Simatos

Pharmacological activation of constitutive androstane receptor induces female-specific modulation of hepatic metabolism

Marine Huillet, Frédéric Lasserre, Marie-Pierre Gratacap, Beatrice Engelmann, Justine Bruse, Arnaud Polizzi, Tiffany Fougeray, Céline Martin, Clémence Rives, Anne Fougerat, Claire Naylies, Yannick Lippi, Géraldine Garcia, Elodie Rousseau-Bacquie, Cécile Canlet, Laurent Debrauwer, Ulrike Rolle-Kampczyk, Martin von Bergen, Bernard Payrastre, Elisa Boutet-Robinet, Laurence Gamet-Payrastre, Hervé Guillou, Nicolas Loiseau, Sandrine Ellero-Simatos

Table of contents

Supplementary methods……………………………………………………………….....2

Fig. S1…………………………………………………………………………………3

Fig. S2…………………………………………………………………………………4

Fig. S3…………………………………………………………………………………5

Fig. S4…………………………………………………………………………………6

Fig. S5…………………………………………………………………………………7

Fig. S6…………………………………………………………………………………8

Fig. S7…………………………………………………………………………………9

Table S1……………………………………………………………………………..10

Table S2……………………………………………………………………………..11

Table S3……………………………………………………………………………..13

Table S4-6…………………………………………………………separate excel files

Supplementary references………………………………………………………..…17

Supplementary methods

1. Trimethylamine-N-oxide targeted LC-MS/MS measurement

For TMAO extraction method from [1] was modified. Liver tissue was combined with 425 µl ice-cold methanol and 75 µl 1% formic acid. Samples were homogenized using a TissueLyser II (30 Hz, 10 min; Retsch Qiagen). After centrifugation (5 min, 8°C, 14.000 rpm) 170 µl of supernatant was transferred to a new tube and evaporated to dryness (Eppendorf concentrator plus, Eppendorf). Prior to measurement, samples were resuspended in LC solvent A:B (1:1), volume depending on the liver weight used for extraction. Of each sample, 10 µl were injected onto an Agilent 1290 II infinity UPLC system (Agilent Technologies Inc., Santa Clara, USA) coupled online to a QTRAP®6500 mass spectrometer (Sciex, Framingham, USA). Chromatographic run was achieved using a reverse phase XSelect HSS T3 XP column (2.1 x 150 mm, 2.5 µm, 100 Å; Waters, Milford, USA) with a binary solvent system 5% acetonitrile with 0.1% formic acid in water (A) and 5% water with 0.1% formic acid in acetonitrile (B). Constant flow rate was set to 250 µl and column oven temperature was at 30°C. Mass spectrometric measurement was performed in positive ionization mode. For identification and quantitation, a scheduled multiple reaction monitoring (MRM) method was used with m/z 76.0 → 59.0 and m/z 75.0 → 42.2 as specific transitions for TMAO. Peak areas were determined in Analyst® Software (v. 1.7.1, Sciex) and exported. Analytical standard trimethylamine N-oxide (TMAO) was purchased from Sigma Aldrich (St Louis, MO, USA). All solvents for MS were of analytical grade purity. Experimental water (resistivity of 18.2 MΩ cm) was purified using a Milli-Q system (Millipore, Milford, MA, USA).

Fig. S1: Impact of CAR activation on hepatic transcripts in *Car*$^{+/+}$ *vs. Car*$^{-/-}$ mice. A) PCA score plots of the whole liver transcriptomic dataset in *Car*$^{+/+}$ and *Car*$^{-/-}$ mice (n=6/group). B) Venn diagram representing the number of genes significantly modulated by TCPOBOP in the liver of *Car*$^{-/-}$ *vs. Car*$^{+/+}$ male and female mice.

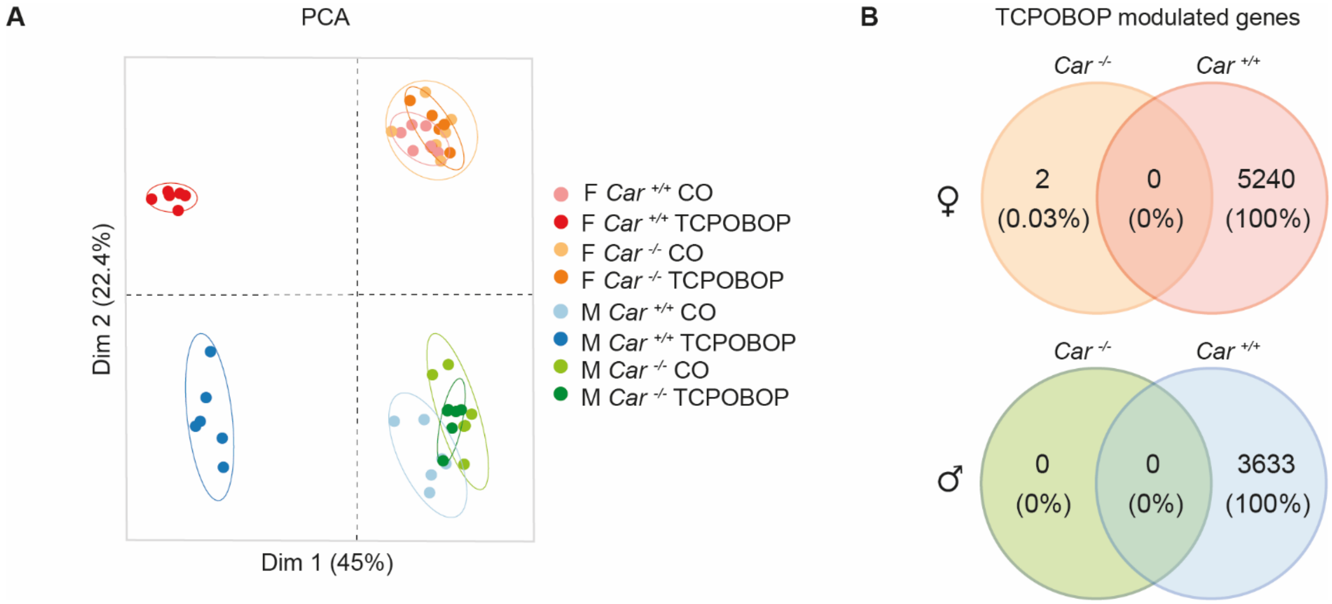

Fig. S2: Majority of TCPOBOP-modulated genes are common between sexes. A) Venn diagram representing the number of genes significantly modulated by TCPOBOP in *Car* $^{+/+}$ male and female mice liver considering significant regulation at P_{adj}<0.05 and 3 different fold-change thresholds (TCPOBOP *vs.* CO). B) Venn diagram representing the number of genes significantly modulated by TCPOBOP in *Car*$^{+/+}$ male and female mouse liver considering significant regulation at P_{adj}<0.01 and 3 different fold-change thresholds (TCPOBOP *vs.* CO).

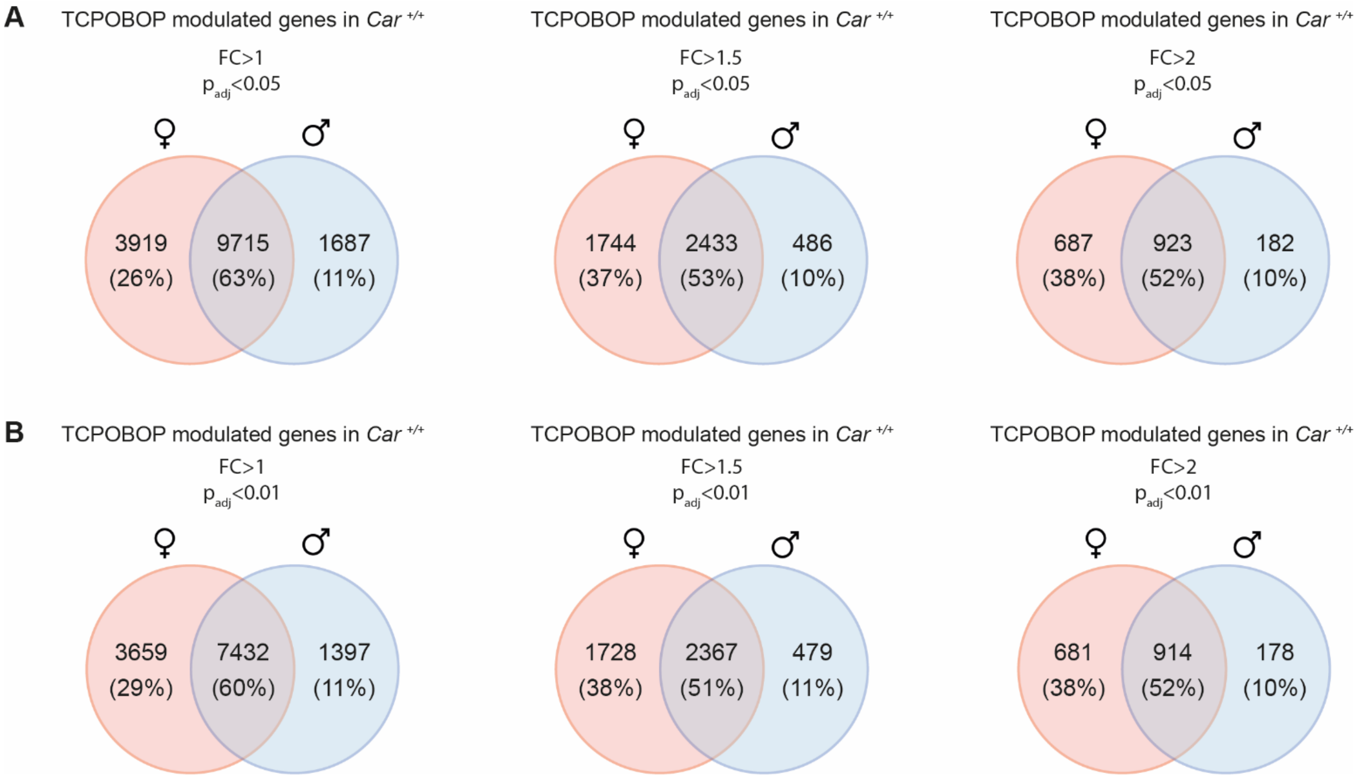

Fig. S3: ^{1}H-NMR-based metabolomics in plasma samples from *Car*$^{-/-}$ mice. A) Coefficient plots related to the PLS-DA models discriminating between ^{1}H-NMR-based plasma spectra from TCPOBOP *vs.* CO *Car*$^{-/-}$ males. Parameters of the PLS-DA model: $Q^2Y=0.1$, $p>0.05$. B) Coefficient plots related to the PLS-DA models discriminating between ^{1}H-NMR-based plasma spectra from TCPOBOP *vs.* CO *Car*$^{-/-}$ females. Parameters of the PLS-DA model: $Q^2Y=-0.4$, $p>0.05$.

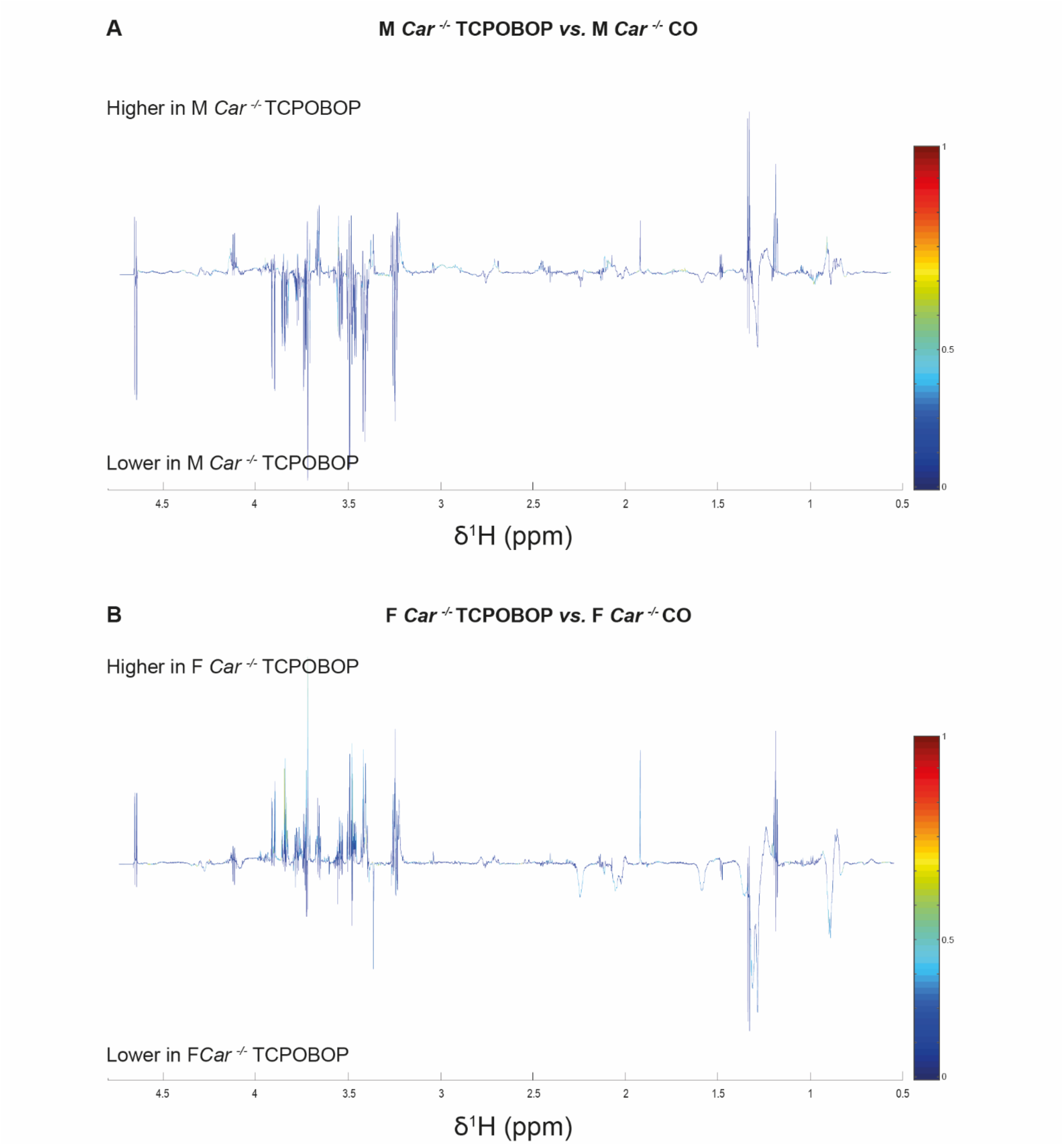

Fig. S4: Effect of CAR activation on plasma biochemistry. A) Plasma cholesterol concentration was determined on plasma collected at the end of the experiment. B) Plasma concentration of high-density lipoprotein (HDL) was determined on plasma collected at the end of the experiment. C) Plasma concentration of triglycerides was determined on plasma collected at the end of the experiment. D) Plasma concentration of free fatty acids was determined on plasma collected at the end of the experiment. E) Plasma concentration of glucose was determined on plasma collected at the end of the experiment. Results are given as the mean ± SEM. *treatment effect, #sex effect. * or # p<0.05, ** or ## p<0.01, *** or ### p<0.001.

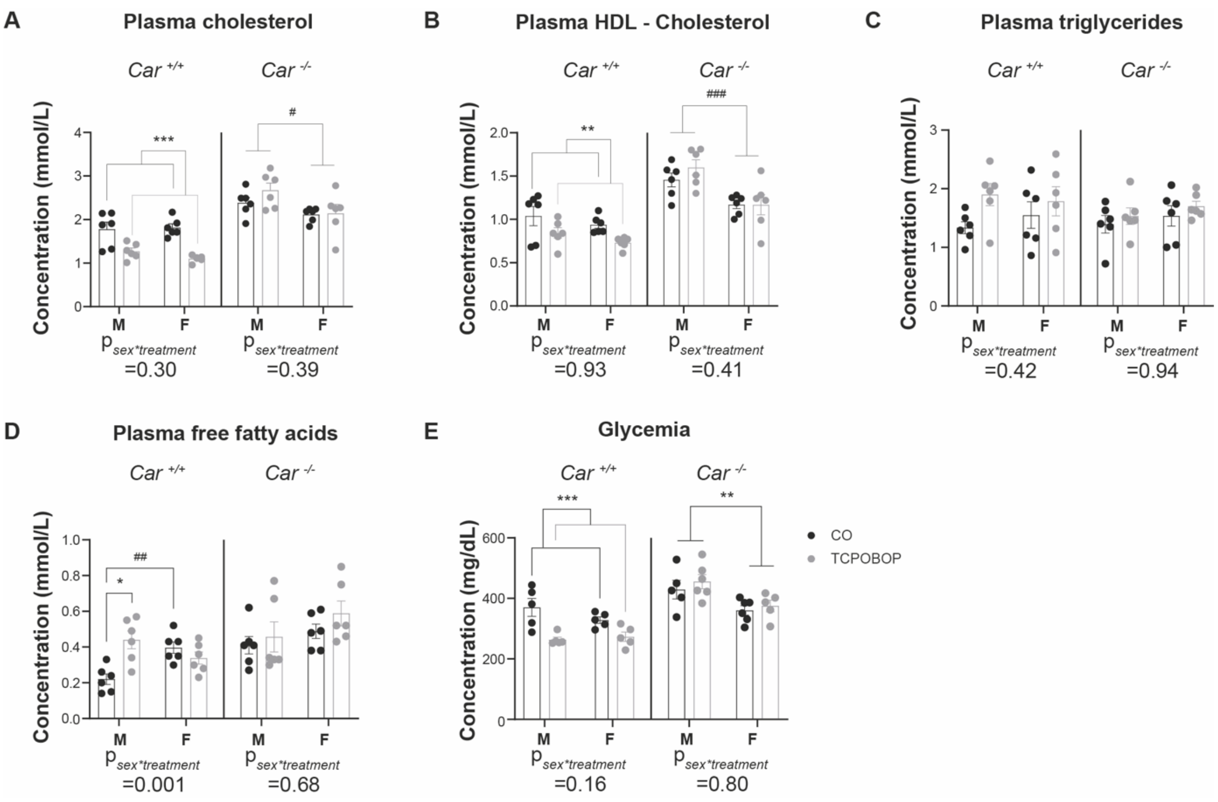

Fig. S5: Effect of CAR activation on hepatic mRNA expression of genes involved in lipoprotein metabolism in *Car*$^{-/-}$ mice. A) Fold-change (TCPOBOP- *vs.* CO-treated *Car*$^{-/-}$ mice) of hepatic expression for genes involved in lipoprotein metabolism (derived from microarray results).

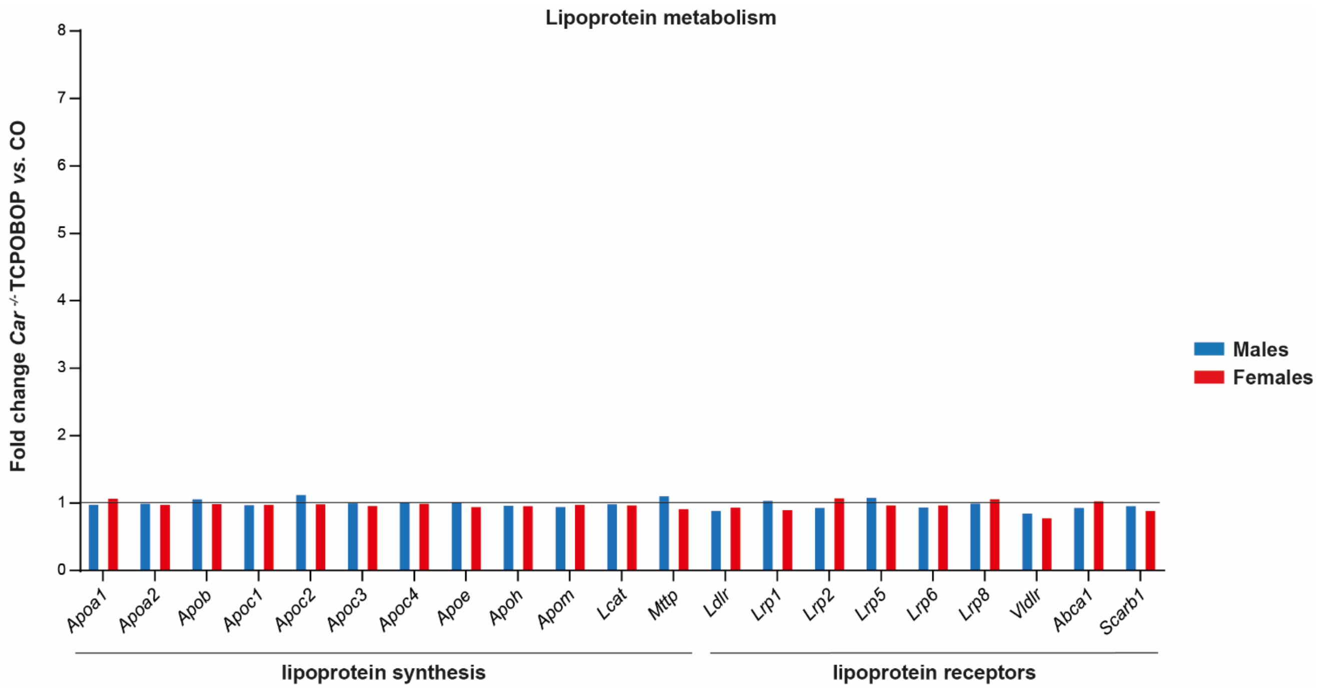

Fig. S6: Pharmacological activation of CAR modulates expression of genes involved in bile acid metabolism and cholesterol metabolism. A) Fold-change (TCPOBOP- *vs.* CO-treated *Car*$^{+/+}$ mice) of hepatic expression for genes involved in bile acid metabolism (derived from microarray results). B) Fold-change (TCPOBOP- *vs.* CO-treated *Car*$^{+/+}$ mice) of hepatic expression for genes involved in cholesterol metabolism (derived from microarray results). C) Fold-change (TCPOBOP- *vs.* CO-treated *Car*$^{-/-}$ mice) of hepatic expression for genes involved in bile acid metabolism (derived from microarray results). D) Fold-change (TCPOBOP- *vs.* CO-treated *Car*$^{-/-}$ mice) of hepatic expression for genes involved in cholesterol metabolism (derived from microarray results). *treatment effect. * $p_{adj}<0.05$, ** $p_{adj}<0.01$, *** $p_{adj}<0.001$.

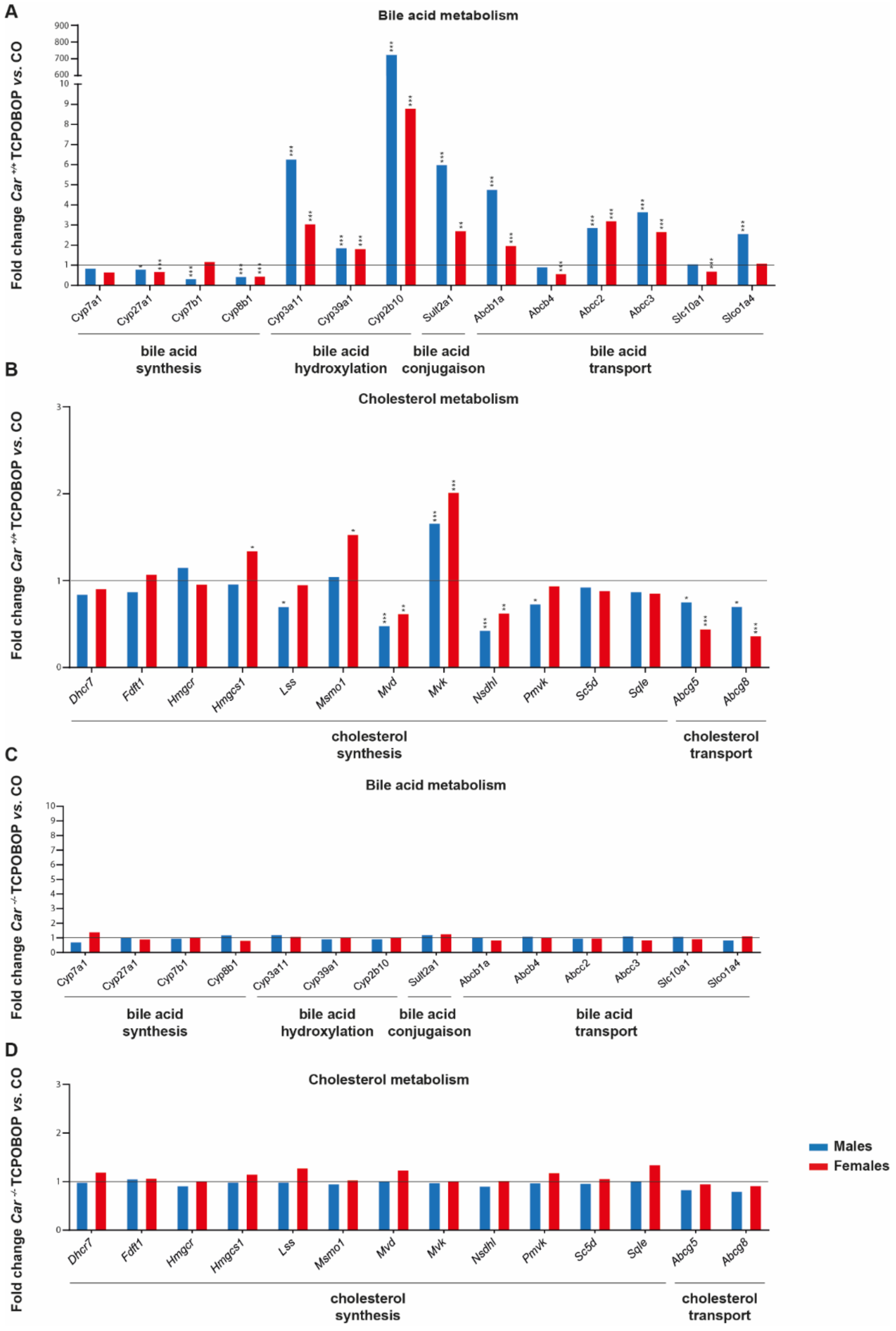

Fig. S7: ^{1}H-NMR-based metabolomics in liver samples from *Car*$^{-/-}$ mice. A) Coefficient plots related to the PLS-DA models discriminating between ^{1}H-NMR-based liver spectra from TCPOBOP *vs.* CO *Car*$^{-/-}$ males. Parameters of the PLS-DA model: $Q^2Y=-0.18$, $p>0.05$. B) Coefficient plots related to the PLS-DA models discriminating between ^{1}H-NMR-based liver spectra from TCPOBOP *vs.* CO *Car*$^{-/-}$ females. Parameters of the PLS-DA model: $Q^2Y=-0.28$, $p>0.05$.

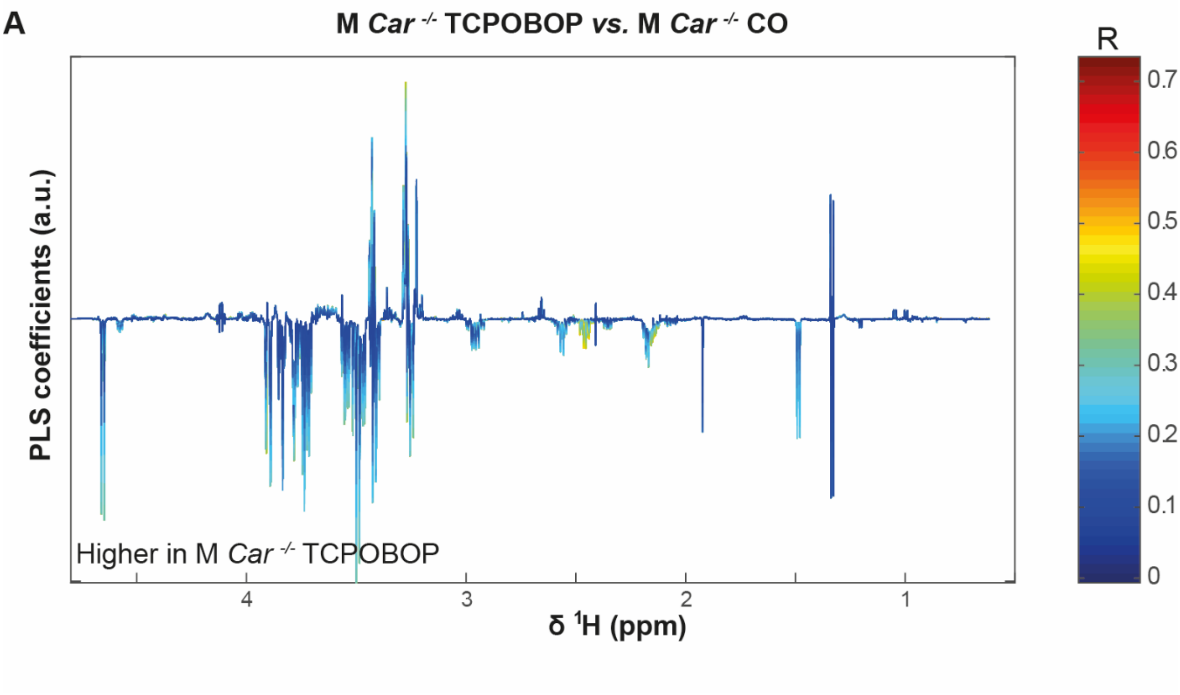

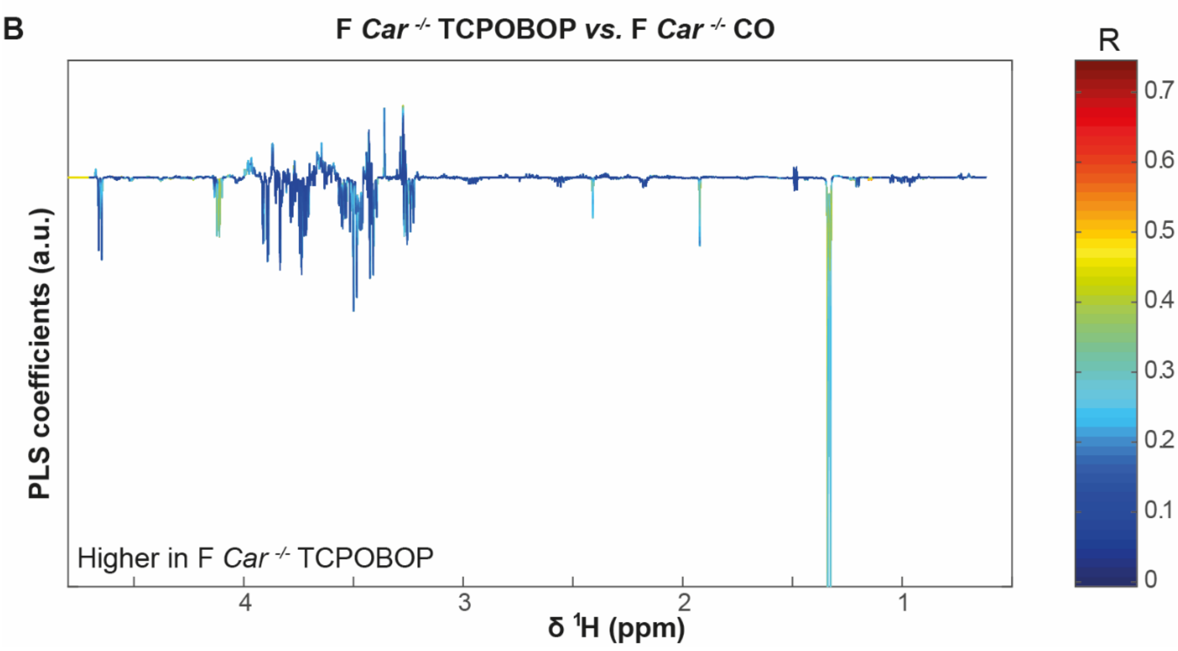

Table S1: Primers used for RT-qPCR.

Gene	NCBI Refseq	Forward primer	Reverse primer
Car	NM_009803	GCTGCAAGGGCTTCTTCAGA	CCTTCCAGCAAACGGACAGA
Cyp2b10	NM_009999	TTTCTGCCCTTCTCAACAGGAA	ATGGACGTGAAGAAAAGGAACAAC
Cyp2c55	NM_028089	TTGTGGAAGAGCTAAGAAAAGCAAAT	GAGCACAGCTCAGGATGAATGT
Fmo3	NM_008030	AAGAAAGGAAGACAAAGAAAAGGCA	AGCTCCAATGATGGCCACTT

Table S2: ^{1}H and partial ^{13}C assignments for identified metabolites in plasma samples. Signals highlighted in bold were used for determination of area under the curves. Keys: s: singlet; d: doublet; t: triplet; q: quadruplet; m: multiplet; dd: doublet of doublet.

x*: tentative assignment

Metabolite	Chemical function	δ ^{1}H (ppm)	Multiplicity	δ ^{13}C (ppm)
Cholesterol (mainly in HDL)		**0.66**	**s (broad)**	
lipid in LDL	$(CH_3(CH_2)_n)$	**0.85**	**s (broad)**	24.6
	(CH2)n	1.23	s (broad)	32.0
lipid in VLDL	$(CH_3CH_3CH_2C{=})$	**0.87**	**t**	16.5
	$CH_2CH_2CH_2CO$	1.29	m	
	CH_2CH_2CO	1.59	m	27.0
Leucine		0.95	t	
Valine		0.99	d	
		1.05	d	20.3
Isoleucine		1.01	d	
Ethanol		1.19	t	19.4
		3.65	q	60.4
lactate		**1.33**	**d**	22.4
		4.12	q	71.3
alanine		1.48	d	19.0
unknown		1.73	m	28.3
acetate		1.92	s	26.0
lipid	$CH_2C{=}C$	2.0	m	29.0
	$CH_2C{=}C$	2.04	m	
methionine		2.13	s	17.1
lipid	CH_2CO	2.24	t	35.7
3-hydroxybutyrate		2.31	dd	
succinate*		2.4	s	
glutamine		2.45	m	33.5
unknown		2.53	s	34.7
methylamine		2.55	s	24.7
unknown		2.69	s	28.5
dimethylamine*		2.71	s	
lipid		2.76	m	28.3
creatine		3.04	s	42.2
glycerophosphocholine		3.22	s	56.7
β-glucose		3.25	m	77.2
		3.47	m	79.0
		3.9	m	63.5
		4.65	d	98.9
α-glucose		3.41	m	72.5
		3.53	m	72.0
		3.71	m	63.0
		3.78	m	63.7
		3.85	m	74.3

	**5.25**	**d**	95.4
unsaturated lipids	5.3	m	132.2
fumarate*	6.52	s	
tyrosine	6.9	d	118.7
	7.19	d	134.7
unknown	7.05	s	
phenylalanine	7.34	m	146.2
	7.43	t	146.4
unknown	7.75	s	147.0

Table S3: ^{1}H and partial ^{13}C assignments for identified metabolites in liver samples. Signals highlighted in bold were used for determination of area under the curves. Keys: s: singlet; d: doublet; t: triplet; q: quadruplet; m: multiplet; dd: doublet of doublet.

Metabolite	δ ^{1}H (ppm)	Multiplicity	δ ^{13}C (ppm)
Bile acids (mixed)	0.6-0.75	s	
	0.92	s	
Bile acids (tauroconjugated)	0.72	s	
	0.92	s	
	3.08	t	
Leucine	0.96	t	23.6
	1.72	m	
	3.74	m	
Valine	0.99	d	19.5
	1.05	d	20.8
	2.29		
	3.62		
Isoleucine	0.94	t	
	1.01	d	
	1.26	m	
	1.48	m	
	2.0	m	
	3.65	d	
3-hydroxybutyrate	1.19	d	24.4
	2.32	dd	
	2.4	dd	
	4.14	m	
Lactate	1.33	d	22.8
	4.11	q	
Threonine	1.33	d	22.3
	3.61		
	4.23		
Alanine	1.49	d	19.1
	3.79		
Ornithine	1.72	m	
	1.93	m	
	3.03	t	41.9
	3.77	t	
Acetate	1.93	s	
L-glutamate	2.06	m	29.8
	2.35	m	36.4
	3.76	dd	
L-glutamine	2.13	m	29.3
	2.45	m	
	3.77	t	
Glutathion (oxidized)	2.17	t	29.1
	**2.53**	**m**	34.2

	2.98	dd	
	3.31	m	41.6
	3.76	m	
	4.75	m	
Glutathion (reduced)	2.17	m	
	**2.56**	**m**	
	2.95	m	
	3.76	m	
	4.56	dd	
Succinate	2.42	s	36.8
Hypotaurine	**2.65**	**t**	
	3.36	t	
L-aspartic acid	2.66	dd	
	2.8	dd	
	3.89	dd	
Dimethylamine	2.72	s	37.6
Trimethylamine	**2.89**	**s**	
Dimethylglycine	2.93	s	46.5
Creatine	3.04	s	39.9
	3.94	s	56.6
Choline	3.2	s	56.7
	3.55		
O-phosphocholine	3.21	s	56.6
	3.58	m	
	4.13	m	
Betaine	3.27	s	55.9
	3.88	s	
Taurine	3.28	t	50.4
	3.44	t	38.5
Methanol	3.36	s	51.7
β-glucose	3.51	m	
	3.75		
	4.66	d	98.7
AMP	4.01	dd	
	4.36	dd	
	4.5	dd	
	6.14	d	89.6
	8.27	s	
	8.62	s	
α-glucose	3.45	m	
	3.56		
	3.72		
	3.84		
	3.97		
	5.25	d	94.8
Glycine	3.56	s	44.4
UDP-glucose	5.6	dd	
	5.95	d	
	7.93	d	

UDP-glucuronate	5.6	dd	
	5.98	d	
	7.96	d	
Uridine	5.89	d	
	5.9	d	
	7.89	d	
NADP+	6.03	s	
	6.1	d	
	8.15	d	
	8.42	s	
	8.584	s	
	8.82	s	
	9.12	d	
	9.3	s	
NAD+	6.04	d	89.5
	6.09	d	102.6
	8.18	s	
	8.2	dd	
	8.44	s	
	8.84	d	
	9.15	d	
	9.34	s	
Inosine	6.11	d	91.1
	8.23	s	
	8.34	s	
Fumarate	6.52	s	
Tyrosine	6.87	d	
	7.19		132.5
Phenylalanine	3.12	dd	
	3.26	dd	
	7.33	m	
	7.38	m	
	7.43	m	
Nicotinurate	3.99	s	
	7.6	dd	
	8.25	m	
	8.72	dd	
	8.94	s	
Nicotinamide	7.61	dd	
	8.23	dd	
	8.7	dd	
	8.95	s	

Table S4: Fold-changes, p-values and pathway enrichment analysis for all TCPOBOP modulated genes (related to Fig. 2c). A) List of genes contained in cluster 1 with their Log(fold-change) values and adjusted p-values in *Car*$^{+/+}$ males and females. B) Gene Ontology (GO) term enrichment analysis using the list of genes contained in cluster 1. C) List of genes contained in cluster 2 with their Log(fold-change) values and adjusted p-values in males and females *Car*$^{+/+}$. D) GO term enrichment analysis using the list of genes contained in cluster 2. Metabolic pathways were considered as significantly enriched when P_{adj}<0.05.

Table S5: Expression of previously-described CAR target genes in both sexes (related to Fig. 2e-g). Table presenting list of genes well-described in the literature as CAR-sensitive genes with their Log(fold-change) values and adjusted p-values in males and females *Car*$^{+/+}$. Data were derived from liver microarray data.

Table S6: Fold-changes, p-values and pathway enrichment analysis for genes modulated by TCPOBOP in a sex-dependent manner (related to Fig. 3b). A) Genes contained in cluster 1 with their Log(fold-change) values and adjusted p-values in *Car*$^{+/+}$ males and females. B) GO term enrichment analysis. C) Genes contained in cluster 2 with their Log(fold-change) values and adjusted p-values in males and females *Car*$^{+/+}$ groups. D) GO term enrichment analysis. E) List of genes contained in cluster 3 with their Log(fold-change) values and adjusted p-values in males and females *Car*$^{+/+}$ groups. F) GO term enrichment analysis. G) List of genes contained in cluster 4 with their Log(fold-change) values and adjusted p-values in males and females *Car*$^{+/+}$ groups. H) GO term enrichment analysis.

Supplementary references

1. Rox, K., Rath, S., Pieper, D. H., Vital, M. & Brönstrup, M. A simplified LC-MS/MS method for the quantification of the cardiovascular disease biomarker trimethylamine-N-oxide and its precursors. *J. Pharm. Anal.* 11, 523–528 (2021).
